# Monitoring soil salinization and waterlogging in the northeastern Nile Delta linked to shallow saline groundwater and irrigation water quality

**DOI:** 10.1038/s41598-024-77954-x

**Published:** 2024-11-13

**Authors:** Mohammed Hagage, Abdulaziz M. Abdulaziz, Salwa F. Elbeih, Abdel Galil A. Hewaidy

**Affiliations:** 1 Engineering Applications and Water Division, National Authority for Remote Sensing and Space Sciences (NARSS), P.O. Box: 1564, El-Nozha El-Gedida, Cairo, Egypt; 2 Faculty of Engineering, Cairo University, Gamaa Street, P.O. Box 12613, Giza, Egypt; 3 Geology Department, Faculty of Science, Al-Azhar University, P.O. Box 11751, Nasr City, Cairo, Egypt

**Keywords:** Archaeological site deterioration, Correlation analysis, Irrigation water quality, Kriging interpolation, Water quality indices, Soil degradation, Spectral water indices, Environmental sciences, Hydrology

## Abstract

Soil salinization and waterlogging are critical environmental issues affecting agricultural productivity and cultural heritage preservation, particularly in arid regions. This study investigated soil degradation processes in the archaeologically and agriculturally significant northeastern Nile Delta of Egypt. The objective was to assess the severity of soil degradation and identify key drivers related to water resources and soil characteristics to aid in the development of management strategies. The research employed a multi-faceted approach, including hydrochemical analyses (of groundwater, irrigation water, and soil), water quality indices calculations, statistical analyses, and satellite data. The results revealed high levels of soil salinization in the northern and central areas, with 64% of soil samples classified as strongly and very strongly saline. Soil chemistry indicated salinization sources linked to sodium chloride dominance. Satellite data from Sentinel-2 images and SRTM digital elevation data showed widespread severe waterlogging in the northern lowlands. The Irrigation Water Quality Index (IWQI) values indicated that 87.5% of irrigation water samples posed severe restrictions due to high salinity and sodium hazards, which were mismatched with the low soil permeability observed in 81% of the collected samples exhibiting clay texture and covering most of the study area. Furthermore, shallow groundwater at depths of 0.5–3 m with high salinity was detected, where total dissolved solids exceeded 20,000 mg/L, and Na-Cl water types prevailed, indicating saltwater intrusion. A strong positive correlation (*r* > 0.83) was found between shallow saline groundwater and soil salinity. The combination of poor irrigation water quality, shallow saline groundwater tables, and low-permeability soils created a synergistic effect that severely compromised soil health and agricultural productivity. It also posed severe risks to the structural integrity of archaeological sites and buried artifacts through accelerated physical and chemical weathering processes. This necessitates an urgent mitigation strategy to combat soil degradation in this critical area.

**Supplementary Information:**

The online version contains supplementary material available at 10.1038/s41598-024-77954-x.

## Introduction

Soil salinization and waterlogging are significant environmental challenges that have far-reaching implications for agriculture, natural ecosystems, and cultural heritage, particularly in arid and semi-arid regions^1–6^. Globally, it is estimated that approximately 20% of the world’s cultivated land and 33% of all irrigated land are affected by soil salinization, and more than 50% of the arable land is projected to be salinized by 2050^7–9^. Similarly, waterlogging poses a significant threat, with an estimated 10–15% of the world’s irrigated land affected by this issue^10,11^. These challenges are particularly prevalent in arid and semi-arid areas, where the combination of limited precipitation, high evapotranspiration rates, and intensive irrigation practices can exacerbate soil degradation processes^2,8^.

The factors contributing to soil degradation are multifaceted and interrelated. Soil salinization can occur naturally through processes like weathering, the existence of shallow saline groundwater, or seawater intrusion into shallow coastal aquifers^12–17^. However, human activities, including inappropriate irrigation methods, insufficient drainage infrastructure, and the utilization of poor-quality irrigation water, can significantly exacerbate the issue^2,18–22^. Similarly, waterlogging can stem from a combination of natural factors like shallow groundwater tables, low-lying topography, and impermeable soil layers, along with human-induced factors such as excessive irrigation, inadequate drainage systems, and land-use changes^12,23,24^. Rising water tables can further intensify soil salinization and waterlogging by facilitating saline groundwater discharge and capillary rise from shallow aquifers^14,25^.

Soil characteristics play a significant role in determining the drainage capacity and salinization risks of soil^26^. Clay-rich soils have higher moisture retention, while coarse sandy soils exhibit greater permeability and leaching ability^15,27^. When irrigating with saline or sodic water, finer-textured, clay-rich soils are more susceptible to structural degradation as sodium ions disperse clay particles, leading to soil dispersion and crusting^28,29^. The combination of soil salinization and waterlogging can have a more severe impact, and soil texture influences the degree of this impact^30^.

In arid, flood irrigation-dependent regions like Egypt, marginal quality irrigation water with high salinity can degrade soils by adding salt loads^16,31,32^. Using low-quality water and wastewater for irrigation under poorly drained conditions can lead to salt accumulation and waterlogging. As a result of repeated irrigations, salts can move downward through the root zone^30,33^. Climate change may exacerbate these issues, further complicating the management of soil salinization and waterlogging^17,34,35^.

The environmental challenges presented by soil salinization and waterlogging are significant. Salinization can diminish soil productivity, inhibit plant growth, and degrade ecosystems, posing a threat to food security and livelihoods^7,36–38^. It is estimated that soil salinization leads to an annual loss of approximately $27.3 billion in crop production globally^39^. On the other hand, waterlogging can result in root asphyxiation, soil compaction, and the proliferation of soil-borne pathogens, ultimately compromising crop yields and soil quality. In severe cases, waterlogging can render agricultural lands unusable, leading to the abandonment of productive areas^10,13^.

To tackle these challenges, a range of techniques have been utilized for monitoring and evaluating soil salinization and waterlogging. Satellite data and Geographic Information System (GIS) techniques have been instrumental in mapping and monitoring the scope and intensity of these issues on regional and global levels^1,20,38,40–43^. Furthermore, field-based approaches, such as soil sampling, groundwater monitoring, and geophysical surveys, offer crucial data for comprehending the fundamental processes and formulating management strategies^6,15,16,23,32,44^.

The northern Nile Delta, renowned for its agricultural and archaeological importance, is particularly susceptible to the consequences of soil salinization and waterlogging^45^. The rising water table and soil salinity pose significant environmental challenges for archaeological heritage, potentially leading to the deterioration of archaeological monuments’ foundations and artifacts buried beneath the surface. This issue is further complicated in the Nile Delta by the rising groundwater table near the surface, facilitating direct interactions between groundwater and archaeological layers, thereby accelerating degradation through chemical and physical weathering processes^46–53^. Furthermore, the impacts of these processes on agriculture in the Nile Delta are severe. Soil salinization and waterlogging can result in reduced crop yields, decreased soil fertility, and the abandonment of once-productive agricultural lands, thereby threatening the livelihoods of farming communities and the region’s food security^54–60^.

While previous studies have contributed significantly to the understanding of soil degradation in the eastern Nile Delta [e.g.,^19,40,41,61,62^], they have limitations in terms of limited spatial coverage, primarily focusing on the southern and central areas of the Eastern Nile Delta. Moreover, these studies have neglected the environmental implications on archaeological sites, and the specific factors that contribute to the deterioration processes related to rising water tables, irrigation water quality, and soil texture have not been thoroughly examined.

This study aims to bridge the knowledge gap by employing an integrated approach that combines hydrochemical analysis (of groundwater, irrigation water, and soil), water quality indices calculations, statistical analyses, and satellite data. The study seeks to provide a comprehensive assessment of soil salinization and waterlogging in the northeastern Nile Delta, with a specific focus on identifying the drivers to help explore management strategies. The objectives of this study are as follows:

1. Evaluate the extent and severity of soil salinization and waterlogging in the northeastern Nile Delta region, with an emphasis on assessing the implications for archaeological sites and soil structure.
2. Investigate the role of irrigation water quality and shallow saline groundwater as key contributors to soil degradation processes.
3. Examine the relationship between soil texture, irrigation water quality, and groundwater dynamics in exacerbating soil salinization and waterlogging.

By identifying specific drivers of soil salinization and waterlogging in the northeastern Nile Delta, our approach enables more targeted and effective management strategies. This is crucial as soil degradation not only threatens agricultural sustainability but also poses significant risks to archaeological preservation, particularly in areas rich in cultural heritage sites. Addressing these interconnected challenges is essential for maintaining both food security and the integrity of historical sites, highlighting the need for integrated management practices that consider environmental and cultural factors.

## Materials and methods

### Description of the study area

**Fig. 1:**
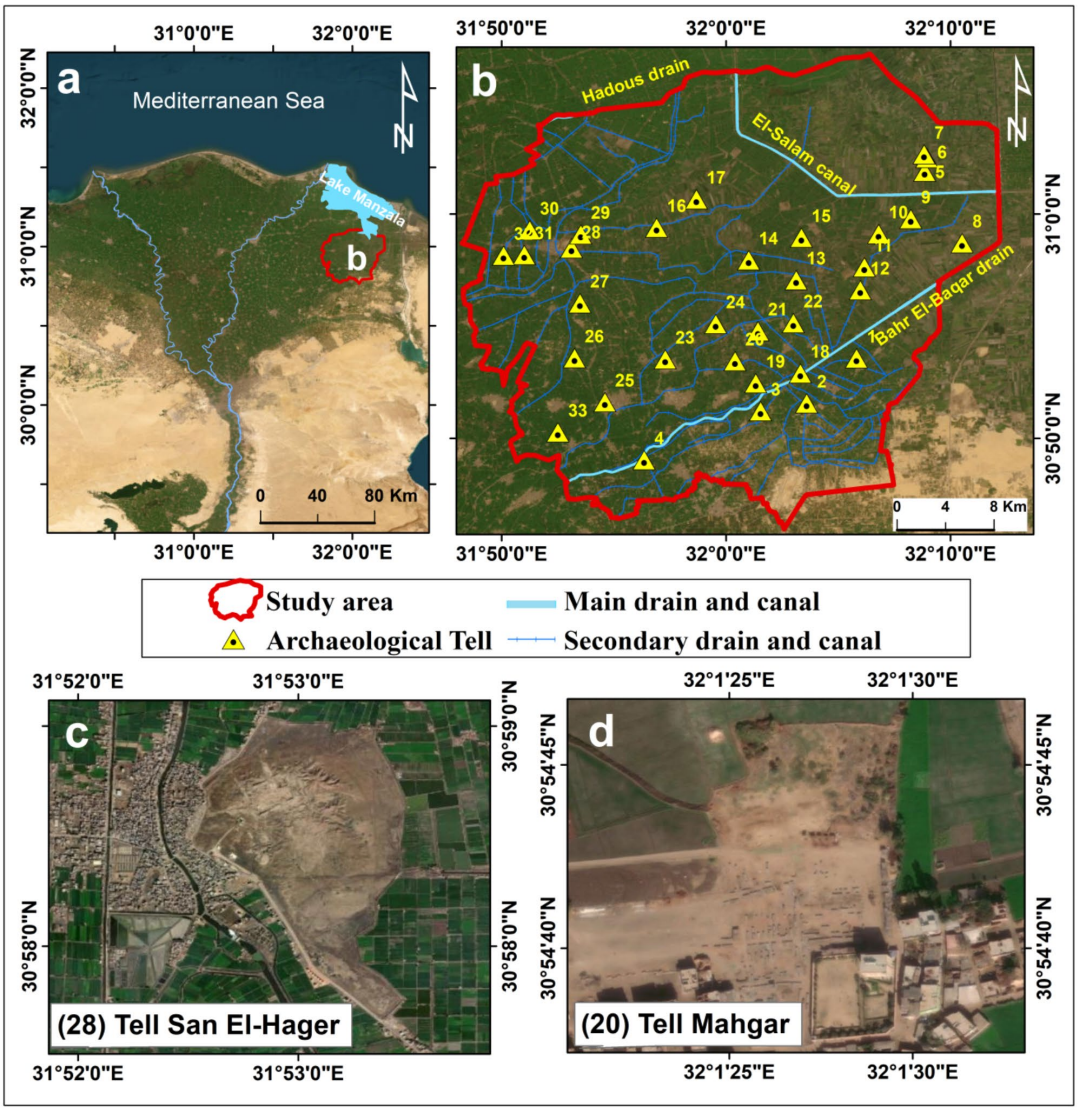
(**a**) Location of the study area within the Nile Delta depicted via satellite imagery (Esri base-map); (**b**) Distribution of archaeological tells and irrigation canals/drains network across the study area; (**c**) Location of Tell San El-Hager (Tanis); (**d**) Location of Tell Mahgar. These maps were created using ArcGIS software version 10.7.1 [https://desktop.arcgis.com].

The study area is situated in the northeastern Nile Delta, south of Lake Manzala, encompassing El-Hosayneya district. It spans an area of 1047 km^2^ and features a dense network of irrigation canals and drains. The primary waterways in the area include the El-Salam canal, Bahr El-Baqar drain, and Hadous drain (Fig. 1a,b). The Bahr El-Baqar drain serves as the main canal for wastewater disposal between Cairo and the northeastern Nile Delta, receiving agricultural, industrial, and sewage effluent^63^. The El-Salam canal carries a blend of Nile Delta drainage water and Nile water^64^.

Historically, the landscape of the study area has undergone significant changes due to natural factors such as sea level fluctuations and land subsidence, which led to the area being covered by seawater and Lake Manzala. Additionally, the landscape has been altered in recent decades following the construction of the Aswan High Dam in the 1960s, along with land reclamation efforts, as the construction of the El Salam Canal helped to confine Lake Manzala behind it. The establishment of dense drainage systems, primarily situated below the water table, has also contributed to lowering the groundwater Tables^45,65^.

The region features an arid climate characterized by very low annual rainfall, typically ranging from 20 to 100 mm, with most precipitation occurring during the winter months. Additionally, the climate is marked by high temperatures, with average daily maximum temperatures ranging from approximately 19 °C in January to exceeding 37 °C during the summer months^66^.

Quaternary sediments of varying sand, clay and gravel proportions, with lateral variations in thickness, cover the area^67^. The main aquifer characterized by sand and gravel with clay lenses within Early Pleistocene deposits^68^. Overlying these are 10–30 m thick Holocene clay, silt and sandy clay deposits that serve as an aquitard^69^. Surface water systems strongly influence groundwater recharge rates, flow direction, and quality^70^. Discharge occurs through pumping for irrigation and domestic use, and through seaward seepage into Lake Manzala and the Mediterranean Sea^71^.

The study area is notable for its substantial agricultural and archaeological significance. Agricultural activities in the northeastern Nile Delta are vital to the local economy, offering employment opportunities and contributing significantly to the country’s agricultural production^72^. In addition, the region has a rich heritage, containing thirty-three archaeological tells spanning the Prehistoric (5500 − 3100 BC) to Byzantine (330–641 AD) periods^45^ (Fig. 1b). Archaeological tells are artificial mounds formed over hundreds or thousands of years of continuous settlement^73^. The most well-known tells in the area are Tell San El-Hager (Tanis) and Tell Mahgar (Fig. 1c,d). Tell San El-Hager (Tanis) was the residence and burial place of 21st-22nd Dynasty kings (1070 − 730 BC), containing remnants of Amun temple and royal tombs^74,75^. Tell Mahgar have cemeteries from the Proto-Dynastic and Early Dynastic periods, as well as a Greco-Roman city and cemetery^76^.

### Field work and laboratory analysis

**Fig. 2:**
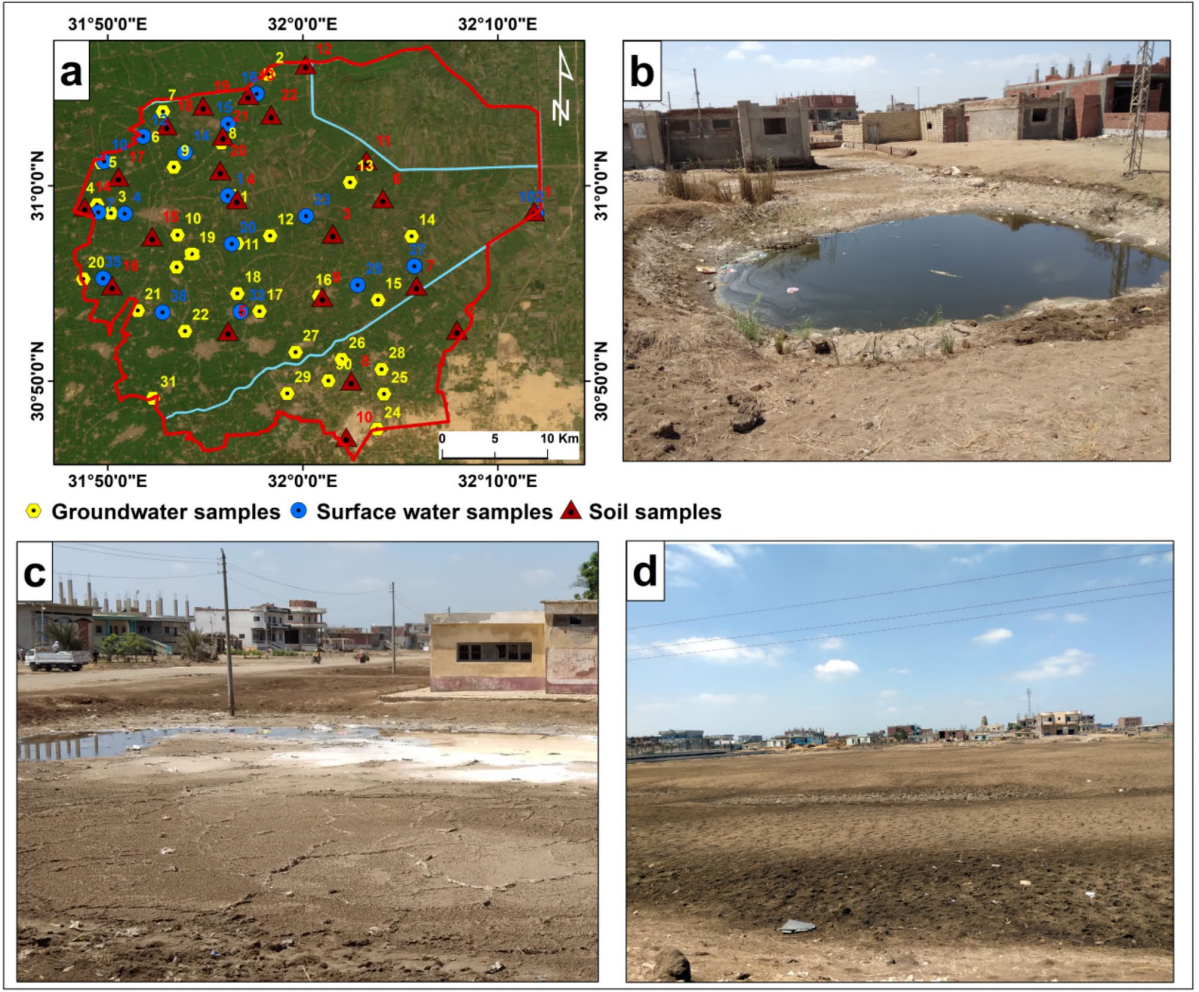
(**a**) Map showing locations of collected surface water, groundwater, and soil samples created using ArcGIS software version 10.7.1 [https://desktop.arcgis.com]. (**b**–**d**) Field photographs depicting soil degradation in the study area due to waterlogging and salinization: (**b**,**c**) Waterlogged urban land with salt crusting on soil surface; (**d**) Abandoned cropland due to salinity.

Groundwater, surface water, and soil samples were collected in May 2023, accompanied by field data collection on the water table and soil degradation across the study area (Fig. 2). Sampling point coordinates were recorded using a Garmin GPS device and mapped using ArcMap Software version 10.7.1^77^. Forty-seven water samples were collected, comprising thirty-one groundwater samples representing the shallow Quaternary aquifer at depths ranging from 5 to 15 m below the ground surface, and sixteen surface water samples from irrigation drains and canals. Twenty-two surface soil samples (0–30 cm depth) were collected to determine their mechanical and physicochemical properties.

Appropriate quality assurance and quality control (QA/QC) measures were diligently applied throughout the sampling, transportation, and laboratory analysis processes to uphold the accuracy and reliability of the analytical data. Field blanks and duplicates were collected at a rate of 10% of the total samples to assess potential contamination during sampling and transportation, as well as to evaluate the precision of the analytical methods. Furthermore, standard reference materials and calibration standards were meticulously used in conjunction with the samples to validate the accuracy of the laboratory instruments and procedures.

Regular calibration of the analytical instruments was conducted, and all water analyses strictly adhered to standardized methods and protocols outlined in APHA^78^ guidelines. Prior to groundwater sample collection, wells were pumped for several minutes to purge stagnant water. Field parameters, including pH and electrical conductivity, were measured on-site using a Solinst interface meter. Samples were filtered through 0.45 μm membrane filters and collected in polyethylene bottles. The bottles were stored in an icebox and transported to the Central Laboratory for Environmental Quality Monitoring (CLEQM) (https://cleqm.nwrc.gov.eg/default.php). Total dissolved solids (TDS) were determined using the gravimetric method^79^, while major cations and anions were analyzed using ion chromatography (IC)^80^.

Particle size distribution of surface soil samples was determined using the pipette method^81^and employed to classify soil texture and permeability based on the USDA soil texture triangle^82^. Furthermore, physicochemical parameters such as pH, electrical conductivity, and major ions were analyzed using standard methods^83–85^.

### Irrigation Water Quality Index (IWQI)

To evaluate irrigation water quality and its relationship to land degradation, the IWQI developed by Meireles et al^86^. was utilized, based on Eq. 1. The IWQI was determined through the following steps: (1) identifying the key parameters that affect irrigation water quality (SAR, EC, Na^+^, Cl^−^, and HCO_2_^−^); (2) calculating the quality measurement values (q_i_) using Eq. 2, based on the irrigation water quality parameter limits established by Ayers and Westcot^87^, as shown in Supp Table 1; (3) establishing the weight values (w_i_) for each parameter, as indicated in Supp Table 1.

1 $$\varvec{IWQI}=\sum_{\varvec{i}=1}^{\varvec{n}}{\varvec{q}}_{\varvec{i}}{\varvec{w}}_{\varvec{i}}$$

2 $${\varvec{q}}_{\varvec{i}}={\varvec{q}}_{\varvec{imax}-}\frac{\left[\left({\varvec{x}}_{\varvec{ij}}-{\varvec{x}}_{\varvec{inf}}\right)\times{\varvec{q}}_{\varvec{iamp}}\right]}{{\varvec{x}}_{\varvec{amp}}}$$

IWQI is a dimensionless parameter ranging from 0 to 100. q_i_ is the quality of the i^th^ parameter, while w_i_ denotes the normalized weight of the i^th^ parameter. q_imax_ corresponds to the maximum value of q_i_ for the class. x_ij_ represents the observed value for the parameter. x_inf_ is the corresponding value to the lower limit of the class to which the parameter belongs. q_iamp_ denotes the class amplitude, and x_amp_ refers to the class amplitude to which the parameter belongs.

The IWQI was classified into five categories indicating the degree of restrictions on water use for irrigation purposes: no restriction (85–100), low restriction (70–85), moderate restriction (55–70), high restriction (40–55), and severe restriction (0–40), based on the obtained index value^86^.

### Groundwater quality indices

The Piper diagram groundwater quality indices, GQI_Piper(mix)_ and GQI_Piper(dom)_, assessed groundwater chemical composition and delineated hydrogeochemical domains based on measured water quality data^88^ (Eqs. 3, 4). GQI_Piper(mix)_ ranges from 0, denoting highly saline water (Na-Cl domain), to 100, signifying highly fresh water (Ca-HCO_3_ domain). GQI_Piper(dom)_ spans 0 to 100, representing Ca-Cl and NaHCO_3_ water endmembers, respectively. The indices ranges and corresponding hydrogeochemical domain classifications are presented in Table 1.

3 $${\text{GQI}}_{{{\text{Piper}}\left( {{\text{mix}}} \right)}} = \left[ {\frac{{\left( {{\text{Ca}}^{{2 + }} + {\text{Mg}}^{{2 + }} } \right)}}{{{\text{Total}}\;{\text{cations}}}} + \frac{{\left( {{\text{HCO}}_{3}^{ - } } \right)}}{{{\text{Total}}\;{\text{anions}}}}} \right] \times 50\left( {in\frac{{meq}}{l}} \right)$$

4 $${\text{GQI}}_{{{\text{Piper}}\left( {{\text{dom}}} \right)}} = \left[ {\frac{{\left( {{\text{Na}}^{ + } + {\text{K}}^{ + } } \right)}}{{{\text{Total}}\;{\text{cations}}}} + \frac{{\left( {{\text{HCO}}_{3}^{ - } } \right)}}{{{\text{Total}}\;{\text{anions}}}}} \right] \times 50\left( {in\frac{{meq}}{l}} \right)$$

**Table 1:** Classifications of groundwater hydrogeochemical domains based on GQI_Piper_index ranges^88^.

Domain	GQI_Piper(mix)_	GQI_Piper(dom)_
Ca-HCO_3_	50–100	25–75
Na-Cl	0–50	25–75
mixed Ca-Na-HCO_3_	25–75	50–75
mixed Ca-Mg-Cl	25–75	25–50
Ca-Cl	25–75	0–25
Na-HCO_3_	25–75	75–100

### Satellite data and spectral indices

Spectral indices are mathematical equations applied on a per-pixel basis to satellite image spectral bands, extracting information about specific Earth surface features or properties^89^. Sentinel-2 Level-2 A surface reflectance data acquired on August 2, 2023 obtained from the Copernicus Open Access Hub (https://scihub.copernicus.eu/) was used to extract waterlogged soil extents across the study area using the Water Index (WI _2015_)^90^. The Sentinel-2 MSI sensor includes 13 spectral bands at 10 m (visible, Near Infra-Red), 20 m (red edge, Short Wave Infra-Red) and 60 m (atmospheric) spatial resolutions^91^.

The level-2 A processing includes a scene classification and an atmospheric correction applied to Top-Of-Atmosphere (TOA) level-1 C orthoimage products, generating an orthoimage atmospherically corrected and surface reflectance product^92^. The Sentinel-2 imagery was processed using the Sentinel Application Platform (SNAP) software, version 9.0.0 (https://step.esa.int/main/download/snap-download/). This processing involved resampling the shortwave infrared band to 10 m, mosaicking, creating subsets, and calculating mathematical equations for the Water Index (WI _2015_). SNAP is free and open-source software developed by the European Space Agency (ESA) specifically for processing and analyzing Earth observation data, with a primary focus on Sentinel satellite imagery^93^.

The Water Index (WI_2015_), developed by Fisher et al.^90^, has demonstrated superior performance compared to other common water indices like MNDWI and NDWI in extracting water bodies from satellite data^94^. The Water Index 2015 is defined as WI_2015_ = 1.7204 + 171ρ_Green_ + 3ρ_Red_ − 70ρ_NIR_ − 45ρ_SWIR1_ − 71ρ _SWIR2_, where ρ represents the surface reflectance for each spectral band. Furthermore, the Shuttle Radar Topography Mission (SRTM) digital elevation data, acquired on February 11, 2000, and obtained from https://dwtkns.com/srtm30m, was utilized to represent land surface elevation in meters above sea level.

### Statistical analysis

Bivariate correlation analysis, employing Pearson’s correlation coefficient, was conducted to assess the relationships between the chemical properties of groundwater and soil. Prior to performing the correlation analyses, the normality of the data was assessed using the Shapiro-Wilk test. All variables satisfied the assumption of normality. Pearson’s coefficient quantifies the strength and direction of a linear association between two variables on a scale from − 1 to 1, where − 1 indicates a perfect negative correlation, 0 signifies no correlation, and 1 represents a perfect positive correlation^95^.

Additionally, spatial interpolation was performed using ordinary kriging in ArcGIS software version 10.7.1 to estimate parameter values at unsampled locations based on measurements from surrounding points. Kriging utilizes statistical models to assess the spatial autocorrelation of the variable, which can then be used to predict values at unsampled locations^96,97^.

## Results and discussion

### Soil degradation and its implications

**Fig. 3:**
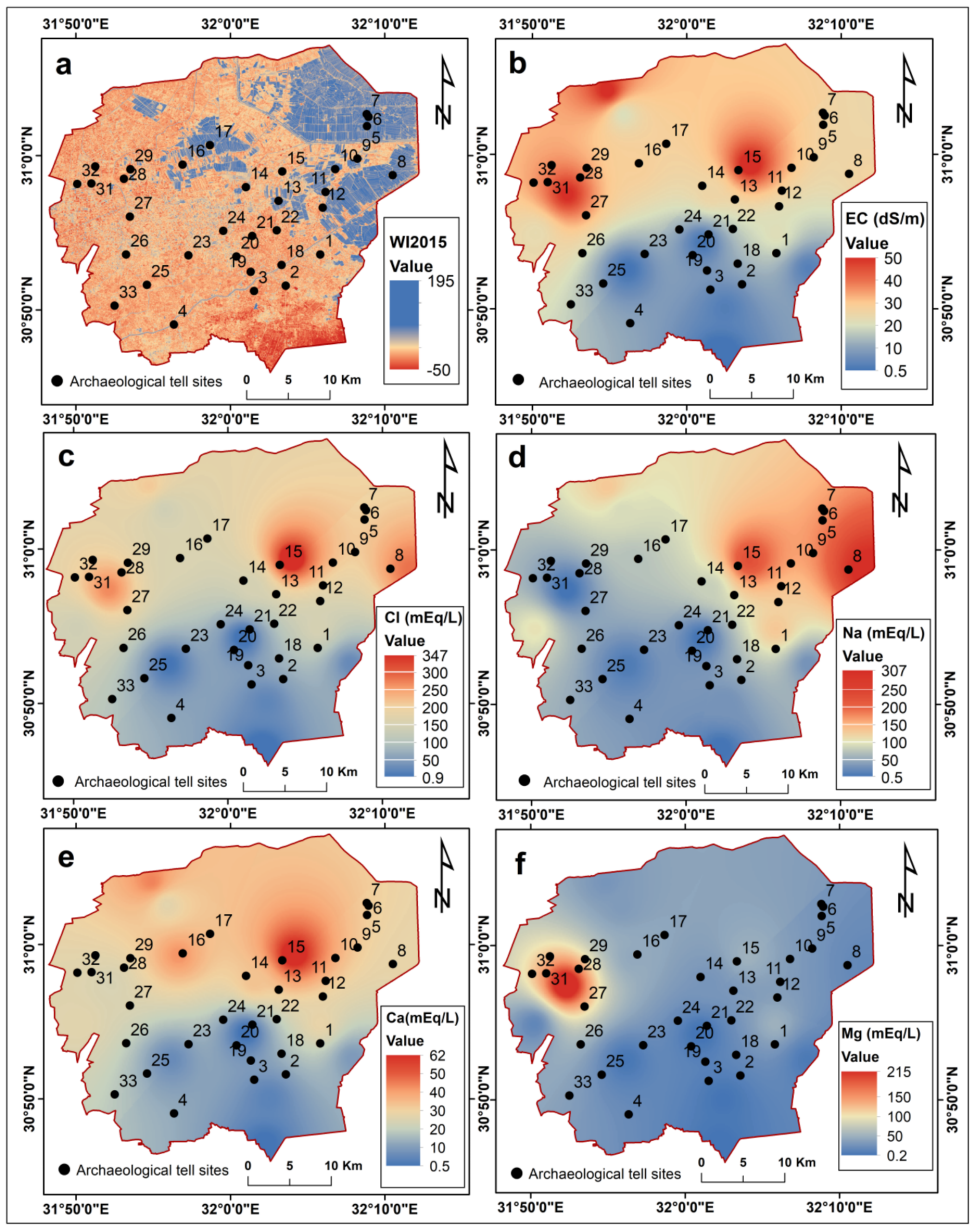
(**a**) Map showing distribution of waterlogged soils based on Water Index (WI) applied to Sentinel-2 A satellite imagery acquired on August 6, 2023. Spatial distribution maps of measured soil chemical parameters: (**b**) Electrical conductivity (EC); (**c**) Sodium (Na^+^); (**d**) Chloride (Cl^−^); (**e**) Calcium (Ca^2+^); (**f**) Magnesium (Mg^2+^). These maps were created using ArcGIS software version 10.7.1 [https://desktop.arcgis.com].

The spatial distribution of soil degradation in the northeastern Nile Delta indicates varying levels of soil salinization and waterlogging (Fig. 3). The map of waterlogged soil and water bodies, generated by applying the Water Index (WI) to Sentinel-2 satellite images, reveals a distinct north-south contrast in index values. The highest WI values are observed in the extensively water-saturated northern soils, in contrast to the lowest values found in the less-saturated southern soils (Fig. 3a). This disparity implies that the northern regions of the study area are more significantly impacted by waterlogging concerns when compared to the southern regions, which exhibit superior drainage capabilities.

The maximum, minimum, and average values of physicochemical properties of the collected soil samples are detailed in Table 2. The pH values exhibit a range from 7.1 to 9.0, with an average of 7.8, indicating a moderately alkaline environment overall. The electrical conductivity (EC) varies between 0.53 and 49.7 dS/m, with an average of 22.2 dS/m. According to^98^, the samples were categorized as follows: 28.5% as non-saline soil (EC < 4 dS/m), 7.1% as slightly saline (4 dS/m ≤ EC < 8 dS/m), 42.8% as strongly saline (16 dS/m ≤ EC < 32 dS/m), and 21.4% as very strongly saline (EC ≥ 32 dS/m). The areas with strongly saline and very strongly saline soils are predominantly concentrated in the northern and central regions of the study area (Fig. 3b).

Furthermore, the soil samples reveal that chloride and sodium are the most concentrated anions in the soil. Chloride content ranges from 0.9 to 348 mEq/L, with a northward increase consistent with the observed pattern in electrical conductivity (EC) (Fig. 3c). Sodium levels exhibit a similar trend, ranging from 0.51 to 307.6 mEq/L, with increasing northward concentrations corresponding to the distributions of EC and chloride (Fig. 3d). In contrast, calcium and magnesium display high variability, ranging from 0.4 to 62 mEq/L and from 0.24 to 215.1 mEq/L, respectively (Fig. 3e,f).

**Table 2:** Summary statistics of physicochemical properties for collected soil samples.

Parameter	Units	Maximum	Minimum	Average	Std. Dev.
pH	-	9	7.1	7.8	0.67
EC	(dS/m)	49.7	0.53	22.2	17.6
Ca^2+^	(mEq/L)	62	0.4	25.6	18.4
Mg^2+^	(mEq/L)	215.1	0.24	44.4	54.7
Na^+^	(mEq/L)	307.6	0.51	95.2	91
K^+^	(mEq/L)	6.9	0.1	1.8	2.1
HCO_3_^−^	(mEq/L)	6.45	0.3	2	1.97
Cl^−^	(mEq/L)	348	0.9	140.8	113.2
SO_4_^2−^	(mEq/L)	78.3	0.625	18.9	23

The results presented indicate high levels of soil salinity and waterlogging in the northern and central parts of the study area. These results are consistent with previous studies conducted in the northern Nile Delta^42,56,57,99–101^, highlighting the widespread and persistent nature of soil salinization and waterlogging issues in this critical area. These earlier investigations employed various techniques to monitor and assess soil degradation. For instance, Abdelaal et al.^99^ evaluated and mapped soil salinity in the northeastern Nile Delta using principal component analysis (PCA), and their results showed that the soil is undergoing an active salinization process. Similarly, AbdelRahman et al.^100^ assessed soil deterioration over the past five decades in the northwestern Nile Delta using satellite data and soil analysis, revealing the prevalence of severe soil degradation processes, including waterlogging, soil compaction, salinization, and alkalization. Furthermore, Abowaly et al.^101^ employed remote sensing and GIS techniques to assess soil degradation north of the Nile Delta, and their results indicated that 83.39% of the area was affected by moderate to high degrees of degradation.

The high levels of soil salinization and waterlogging in the northern and central parts of the study area have significant implications for soil structure and the integrity of archaeological tells. Many archaeological tells are situated in the northern regions, where highly waterlogged soil can severely impact their structural integrity. Continual wetting and drying cycles can weaken bricks and accelerate the dissolution of binding mortar, while erosion can undercut foundations, causing subsidence or collapse^4,102^. Furthermore, biochemical weathering processes like oxidation and organic growth are amplified, irreversibly altering artifacts. Biological weathering, caused by the activity of plants, animals, and microorganisms, involves the weakening and disintegration of rocks and dissolution of minerals^47,103^.

Alkaline soils can pose multiple risks to the physico-mechanical characteristics of the soil, in addition to impairing soil microbes, slowing organic matter decomposition, and making the soil prone to erosion^104,105^. The high salinity can cause disruption of soil structure through clay dispersion and physical weathering of archaeological building materials and decorative elements by salt crystals^29,51^. The high levels of chloride contribute to soil salinity, which affects soil texture and fertility. Chloride can lead to corrosion and pitting on the surface of archaeological stones^106,107^. Furthermore, high sodium levels, especially in the northeastern region, can lead to soil dispersion, reduced water infiltration, and increased runoff and soil erosion^28^.

Unlike sodium, calcium and magnesium levels in soils are crucial for maintaining soil structural stability by binding particles together^108^. Under high sodium conditions in the northeastern region, calcium and magnesium ions are readily displaced from cation exchange sites, leaving soils depleted in these structural stabilizers. This can cause clay dispersion, leading to the sealing off of macropores essential for drainage and oxygen diffusion^109^. This cation displacement explains the over 2 to 3-fold difference between average sodium, magnesium and calcium content in these soils. The resulting sodic soil conditions, characterized by poor structure, low infiltration, and poor aeration, can lead to moisture accumulation, waterlogging, and accelerated weathering, ultimately impacting agricultural productivity^54,55,110^. Additionally, these soil properties can reduce the ability of the land to support the weight and structural integrity of archaeological sites, leading to the accelerated deterioration of archaeological materials and features^50,51,53,111,112^.

The petrographic and mineralogical analyses conducted on artifacts from the archaeological tells within the study area have confirmed the origins of deterioration attributed to waterlogging and soil salinity. The granite obelisks at Tell San El-Hager exhibited various forms of damage, such as scaling and cracking. The primary sources of deterioration were identified as chloride and sulfate salts^52,75^. Moreover, investigations into the building materials sourced from the royal tombs at Tell San El-Hager unveiled the prevalence of halite salts (NaCl) and gypsum (CaSO_4_·2H_2_O) as predominant components. These salts have contributed to the loss of cohesion within the structures due to salt crystallization processes, leading to corrosion^50^.

Soil salinization and waterlogging issues in the northeastern Nile Delta region not only affect archaeological sites and soil structure but also present a significant economic challenge due to their impact on agricultural productivity^13,40,56–58^. The decline in agricultural productivity resulting from soil degradation not only affects local farmers and communities but also has broader economic implications for the region. As the fertile lands in the Nile Delta deteriorate, their current and potential productivity declines, limiting the ability to sustain the growing population and affecting the agricultural sector, which is a vital part of the region’s economy^31,60^. Furthermore, the economic consequences of soil degradation extend beyond the agricultural sector, influencing food security, livelihoods, and overall economic stability in the Nile Delta. Therefore, identifying the causes of soil degradation and implementing appropriate remedial measures is essential to mitigate these economic impacts and preserve the archaeological heritage in the region.

### Driving factors of soil salinization and waterlogging

#### Irrigation water quality and soil texture

The results of the chemical analysis of water samples collected from irrigation canals are presented in Table 3. The pH of the water samples ranged from 7.5 to 7.9 with an average of 7.7, indicating near neutral to slightly alkaline conditions. The electrical conductivity (EC) of irrigation water ranged from 1011 to 4480 µs/cm, with an average of 2611 µs/cm. Total dissolved solids (TDS) ranged from 647 to 2860 mg/L, averaging 1670 mg/L. EC and TDS are critical indicators of salinity risk to soils^113^. According to FAO^114^ guidelines, 31% of samples were classified as very highly saline, with EC exceeding 3000 µs/cm and TDS over 2000 mg/L. The use of this highly saline water poses severe restrictions, as they are likely to rapidly induce salinization and degradation of soils^44^. The remaining 69% of samples had moderate to high salinity, with EC from 700 to 3000 µs/cm and TDS 450 to 2000 mg/L. While less saline than the most degraded samples, this water still poses moderate restrictions on use for irrigation^114^. Over the long-term, the continued use of irrigation water with EC and TDS in this range carries risks of gradual accumulation of salts in the soil profile^115^.

The irrigation water exhibited high sodium levels, with concentrations ranging from 155 to 740 mg/L and sodium adsorption ratio (SAR) values between 4 and 12.5 meq/L. According to Ayers and Westcot^30^, 31% of samples exceeded the SAR threshold above which severe use restrictions apply due to dispersion and permeability impacts. The remaining 69% fell into the medium sodium hazard category, with moderate use restrictions. Prolonged irrigation with this water could still induce issues with water infiltration and drainage^116^. When introduced to soils, sodium ions displace Ca and Mg, deteriorating soil structure to the point of unsuitability for filtration and nutrient uptake^117^. Sodium impacts infiltration by degrading soil structure, especially in surface layers. As sodium accumulates, it causes clay particles to detach and disperse, clogging pores and reducing the rate of water infiltration into the soil^28^.

The impact of sodium-induced dispersion on soil health and agricultural sustainability is concerning, as it can lead to problems including ponding, runoff, waterlogging, poor seedling emergence, reduced aeration, and increased disease pressure^21,118–120^. Additionally, sodium-induced dispersion makes irrigation management more difficult by hindering adequate water supply for crop needs^121^. This issue is exacerbated when combined with salinity hazards, posing a substantial long-term threat to soil health and agricultural sustainability in affected regions^7,122^.

Furthermore, the bicarbonate content of the irrigation water ranged from 331 to 600 mg/L, averaging 448.6 mg/L. This spans the severely restricted (> 500 mg/L) and moderately restricted (90–500 mg/L) categories for irrigation suitability according to FAO^114^ guidelines. The high bicarbonate levels found in many of these water samples are concerning. As water evaporates during irrigation, bicarbonate ions can precipitate out as sodium carbonate, increasing soil pH and sodium adsorption ratio (SAR) over time^123^. High bicarbonate concentrations can also extract calcium from soil particles and sodium replaces calcium in the soil. This further elevates soil sodium levels while reducing soil permeability^124^.

The deterioration of irrigation water quality in the study area is attributable to the use of wastewater after mixing with Nile water, resulting from decreased freshwater supplies^125,126^. Wastewater includes return flows to water bodies, drains, and sewage systems from irrigation, municipal, and industrial activities^127^. Additionally, discharge of sewage into canals and drains exacerbates the degradation, due to lacking sewage networks in some areas. Other contributing factors are runoff of agricultural fertilizers, and release of domestic and industrial wastes from factories^71,128,129^.

To understand the suitability of the irrigation water quality to soil texture, the Irrigation Water Quality Index (IWQI) was calculated (Table 3). The IWQI results indicated that a significant portion of the water (87.5%) falls into the severe restriction category (IWQI = 0–40), suggesting it should not be used for irrigation under normal conditions. If used, the soil must have extremely high permeability^86,130^. Additionally, 6.25% of the water was classified as high restriction (IWQI = 40–55), requiring high permeability soils to avoid salinization and reduce seepage^131^. The remaining 6.25% was classified as moderate restriction (IWQI = 55–70) and performs best in soils with moderate to high permeability^132^.

**Table 3:** Chemical analysis of irrigation water samples and computed SAR and IWQI values.

NO	pH	EC (µs/cm)	TDS (mg/L)	HCO_2_^−^ (mg/L)	Ca^2+^ (mg/L)	K^+^ (mg/L)	Mg^2+^ (mg/L)	Na^+^ (mg/L)	Cl^−^ (mg/L)	SO_4_^2−^ (mg/L)	SAR	IWQI
1	7.5	4120	2630	414	178.68	15	57.63	650	1065.8	319.7	10.81	16.45
2	7.5	2760	1758	448	125.11	9.5	54.04	462	299.5	128.4	8.68	35.70
3	7.6	2790	1780	444	139.54	17	45.97	585	602	266.1	10.97	23.75
4	7.6	1880	1213	497	91.42	9.3	40.72	310	393.6	186.2	6.77	29.05
5	7.7	1840	1185	536	89.66	13	35.18	284	329.9	145.4	6.43	29.83
6	7.6	2120	1338	439	98.64	10	41.69	325	426.9	154.7	6.91	29.19
7	7.6	3340	2140	600	191.03	12	26.14	515	756.9	227.9	9.26	8.40
8	7.9	1880	1207	468	83.88	9	42.67	265	333.3	132.9	5.87	38.25
9	7.5	4440	2840	497	148.04	9.7	69.10	740	1211.7	288.1	12.58	9.45
10	7.7	1770	1132	331	73.62	10	44.22	270	386.5	138.9	6.14	33.21
11	7.7	4480	2860	448	240.6	21	36.93	655	1401	353.6	10.37	16.08
12	7.9	1890	1210	419	98.16	19	30.52	254	353.4	180.4	5.73	38.42
13	7.8	1011	647	361	63.03	7.4	24.49	155	120	74	4.19	67.57
14	7.6	1770	1136	448	96.72	7.8	39.36	232	297	179.4	5.02	47.48
15	7.7	2120	1347	400	107.78	6.08	36.25	320	371	190	6.80	34.90
16	7.5	3310	2131	394	118	7	68.1	395	671	278	7.16	19.60
Max	7.9	4480	2860	600	240.6	21	69.1	740	1401	353.6	12.58	67.57
Min	7.5	1011	647	331	63.03	6.08	24.49	155	120	74	4.19	8.4
Average	7.6	2611	1670	448	124.8	11.66	43.7	406	585.5	203.9	7.8	30.74

**Fig. 4:**
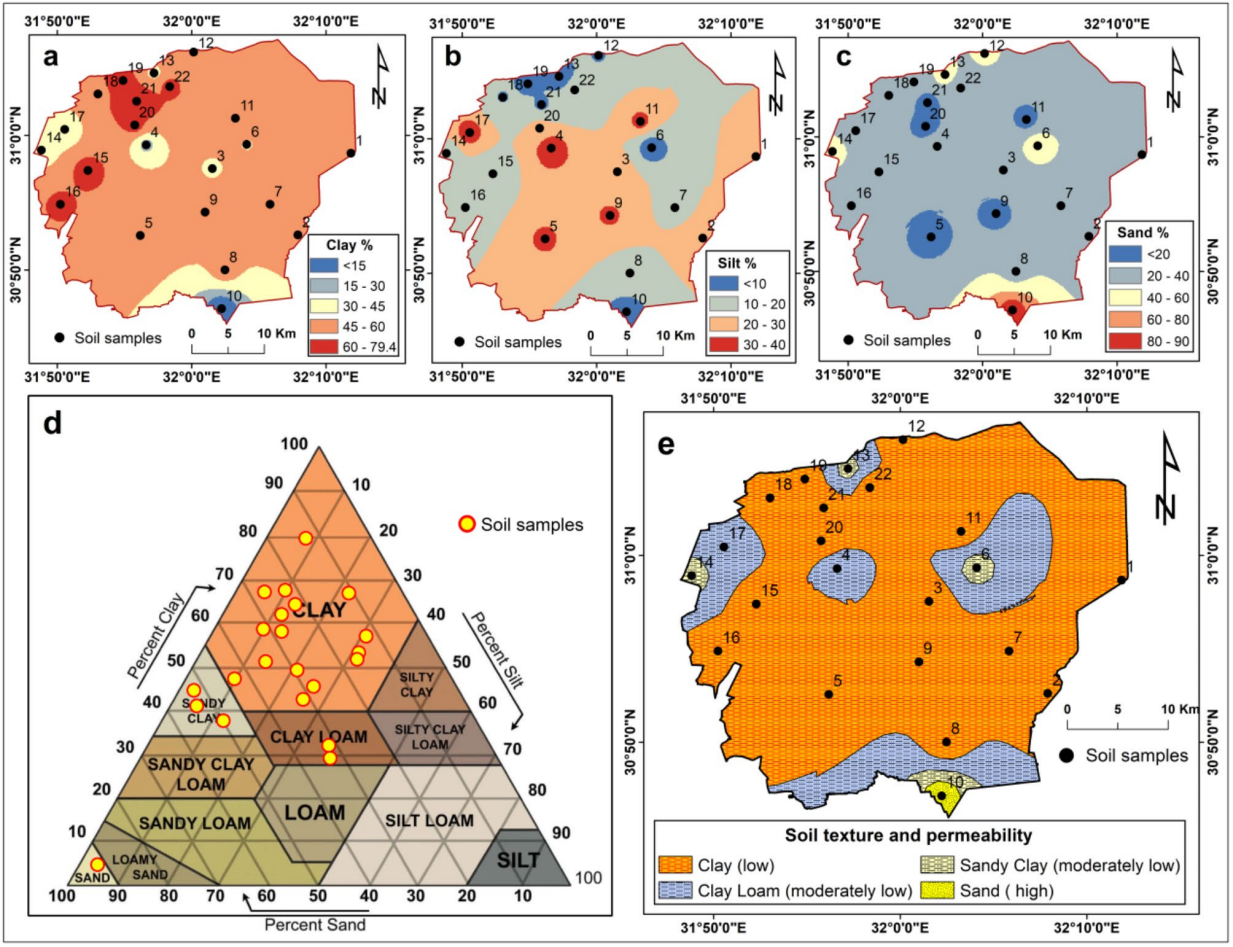
Spatial distribution of mechanical properties of collected soil samples: (**a**) Clay content (%); (**b**) Silt content (%); (**c**) Sand content (%); (**d**,**e**) Soil texture and permeability based on USDA classification.

Based on the IWQI values, the irrigation water requires very high permeability soils to prevent degradation. However, as evidenced by the data in Supp Table 2; Fig. 4, the collected soil samples were predominantly clay-textured with low permeability. The samples were overwhelmingly clay-dominated, with clay content averaging 50% (Fig. 4a). Silt and sand fractions averaged just 18% and 33%, respectively (Fig. 4b,c). When plotted on the USDA texture triangle (Fig. 4d), 81% samples classified as clay, clay loam.

This clay-rich texture results in very slow infiltration and drainage due to small pore size^110^. As shown in Supp Table 2,* 16* of 22 samples had slow permeability, and 5 had moderately permeability. These impermeable soils cover most of the study area (Fig. 4e), presenting a mismatch with the poor-quality irrigation water.

The low permeability of the clay means salts and sodium will readily accumulate, leading to salinization, reduced infiltration, poor drainage, waterlogging, and other issues^2,133^. Therefore, it will be extremely challenging to manage the poor quality water on these soils, even those classified as moderately restricted. The clay soils lack the high permeability needed to leach salts and sodium to prevent degradation.

According to the findings, the severe deterioration of irrigation water quality, along with its incompatibility with soil characteristics, is a significant factor contributing to soil salinization and waterlogging in the study area. This aligns with numerous studies conducted in Egypt and globally, highlighting the pivotal role of irrigation water quality in exacerbating soil degradation [e.g.,^2,38,26,32,20,33,63,44,133^]. Amer^20^ assessed the spatial relationship between irrigation water quality, soil salinity, and waterlogging in the western Nile Delta. The findings demonstrated a significant spatial correlation among soil salinization, waterlogging, and EC, SAR, and sodium levels in irrigation water. Prolonged irrigation with high-salinity water resulted in increased soil salinity, low soil permeability, and waterlogging. Yuan et al^33^. evaluated the impact of saline water irrigation on soil salt distribution and physical properties in the Shiyang River basin of Northwest China. It was observed that higher salinity levels in irrigation water and extended exposure to saline irrigation led to increased salt accumulation and higher soil bulk density.

#### Shallow saline water table

**Fig. 5:**
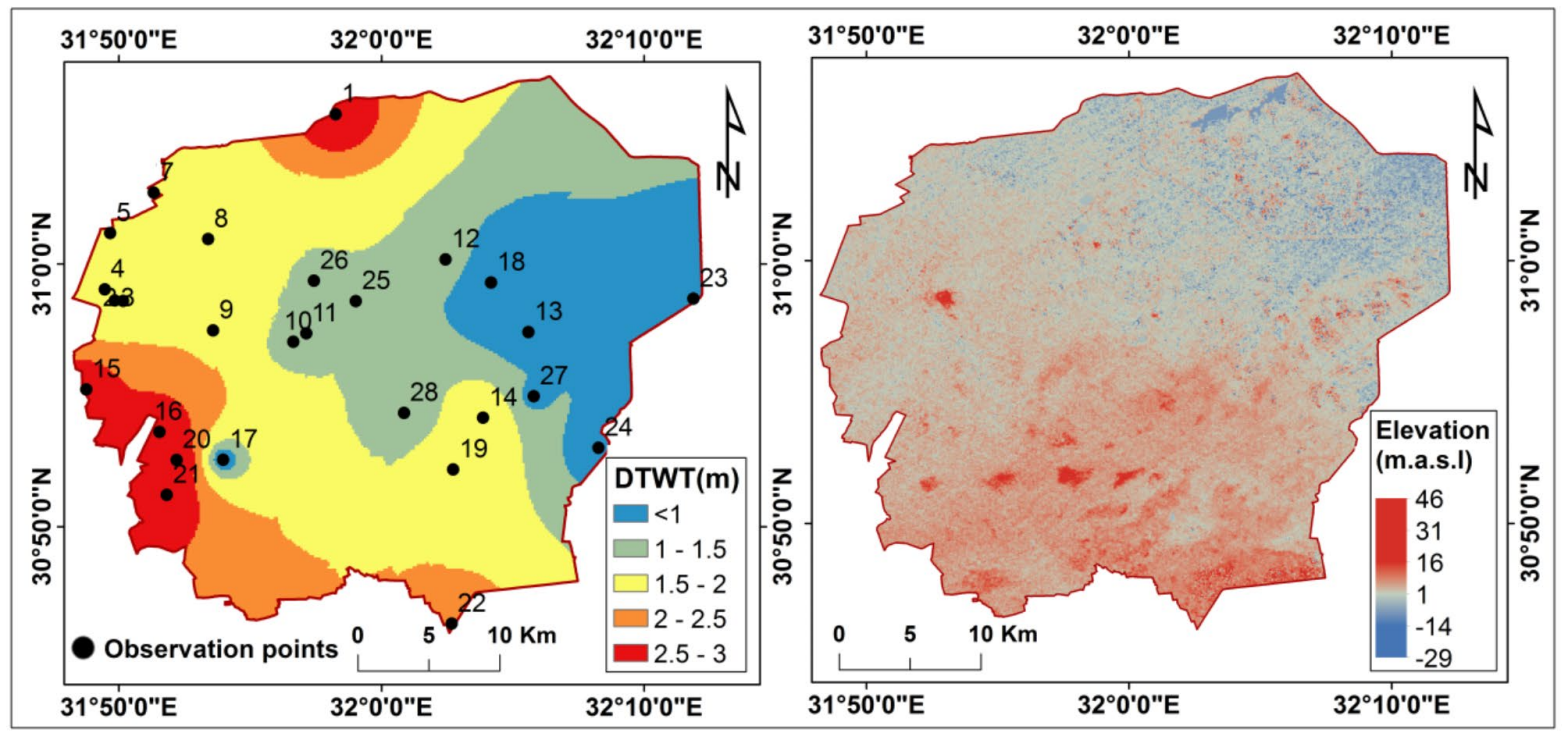
(a) Map showing depth to water table (DTWT) in meters below ground level; (b) Digital elevation model (DEM) showing land surface elevation in meters above sea level derived from SRTM data. These maps were created using ArcGIS software version 10.7.1 [https://desktop.arcgis.com].

The results of the field observations revealed a shallow water table in the study area, with depths ranging from < 0.5 to 3 m below ground level. The shallowest water tables were found in the northeastern region (Fig. 5a), likely due to several interconnected factors:

- *The area’s topography*: The topographic analysis using SRTM DEM data showed the land surface slopes toward the north, with the lowest elevations in the northeast (Fig. 5b). This directly corresponds to the regions with groundwater depths of < 0.5 m.
- *The area’s history*: The northeastern Nile Delta was submerged under seawater between 7000 − 3000 BC. Later, Lake Manzala formed between 600 and 1000 BC, covering the area. After the construction of the Aswan High Dam in the 1970s and subsequent agricultural reclamation, the lake shrank significantly^45,134^. However, the legacy of these historical flooding events remains evident; the high water table is a remnant effect of the former inundation.
- *The fish farms and flood irrigation*: The spread of fish farms, flood irrigation, and poor subsurface drainage systems have exacerbated the high water Tables^40,135^.
- *Land subsidence*: The high subsidence rates of 5–8.4 mm/year recorded in the northeastern Delta^136^ have effectively brought the groundwater even closer to the surface over time.
- *The presence of subsurface clay layers*^69^ with low permeability near the surface create localized shallow water tables when drainage is poor.

**Fig. 6:**
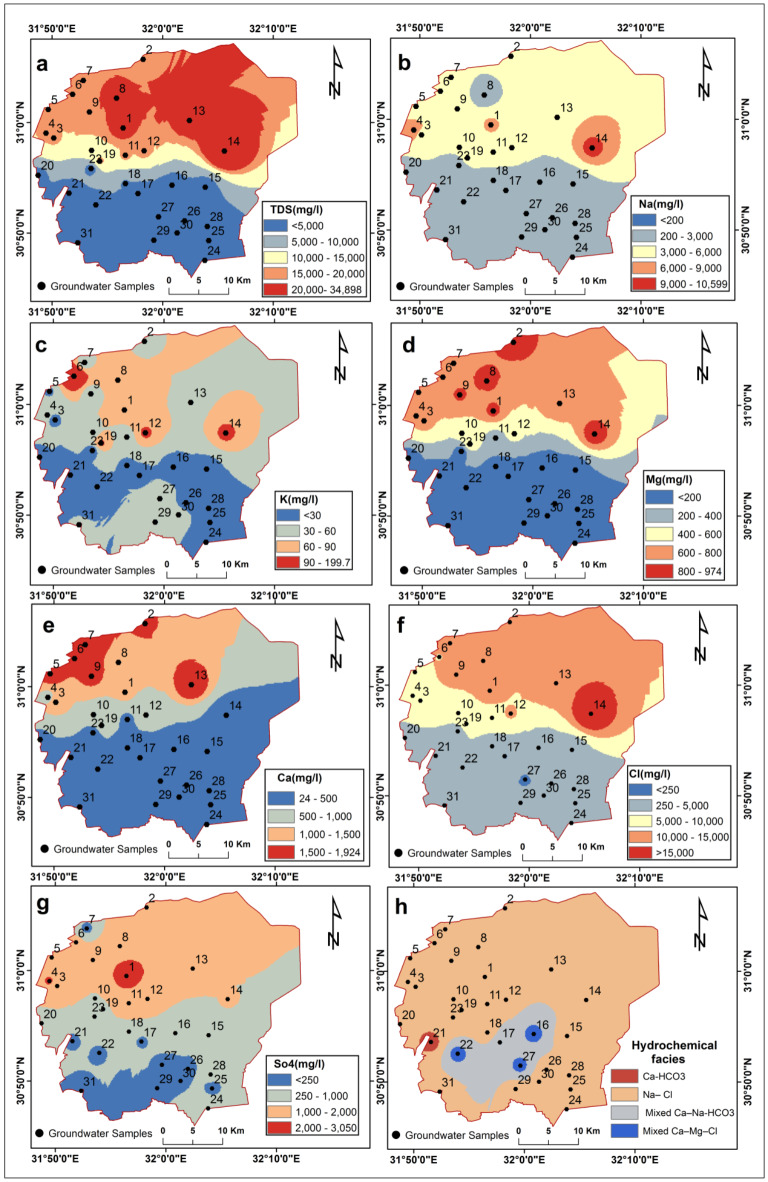
Spatial distribution maps of groundwater chemical parameters and hydrochemical facies: (**a**) Total dissolved solids (TDS); (**b**) Sodium (Na^+^); (**c**) Potassium (K^+^); (**d**) Magnesium (Mg^2+^); (**e**) Calcium (Ca^2+^); (**f**) Chloride (Cl^−^); (**g**) Sulfate (SO_4_^2−^); (**h**) Hydrochemical facies. These maps were created using ArcGIS software version 10.7.1 [https://desktop.arcgis.com].

Summary statistics of hydrochemical parameters of collected groundwater samples compared to WHO^137^and USEPA^138^ standards are shown in Table 4. Spatial distribution maps of total dissolved solids (TDS), sodium, potassium, magnesium, calcium, chloride, sulfate, and hydrochemical facies are shown in Fig. 6a–h, respectively. The groundwater quality revealed high salinity across the study area, with TDS ranging from 837 to 34,900 mg/L (average 10,473 mg/L). TDS exceeded 20,000 mg/L in the northern regions (Fig. 6a). Sodium was the dominant cation at 115 to 10,600 mg/L (average 2821 mg/L), with concentrations spiking over 3000 mg/L in the northern and central regions. This aligns with the spatial distribution of salinity (Fig. 6b). Chloride was the dominant anion at 142 to 25,512 mg/L (average 6370 mg/L), also highest in the north (Fig. 6f). Sulfate was the second most abundant anion at 37.5 to 3052.7 mg/L (average 889.9 mg/L), while calcium was the second most abundant cation at 24 to 1924.8 mg/L (average 650 mg/L).

The dominance and spatial correlation of Na^+^ and Cl^−^ in the north, along with the spread of Na-Cl water types in most of the study area (Fig. 6h), indicate that groundwater is significantly impacted by saltwater intrusion, with saltwater intrusion being the primary source of high salinity in the groundwater. This finding is consistent with previous studies that have confirmed the encroachment of saltwater into the Nile Delta aquifer up to 70 km inland along the aquifer’s bottom boundary in the northern Nile Delta, which has an extensive coastline along the Mediterranean Sea [e.g.,^69,139–143^]. These studies have attributed the exacerbation of saltwater intrusion to a combination of natural factors and inadequate water management practices, including excessive groundwater extraction and rising sea levels.

**Table 4:** Summary statistics of hydrochemical parameters for collected groundwater samples.

Parameter	Maximum	Minimum	Average	WHO^137^	USEPA^138^
TDS (mg/L)	34,900	837	10,473	1000	500
Ca^2+^ (mg/L)	1924.8	24.06	650	-	-
K^+^ (mg/L)	200	5.8	47	-	-
Mg^2+^ (mg/L)	974.43	31	375	-	-
Na^+^ (mg/L)	10,600	115	2821	200	-
Cl^−^ (mg/L)	25512.8	142	6370	250	250
SO_4_^2−^ (mg/L)	3052.7	37.5	889.9	250	250
HCO_2_^−^ (mg/L)	1118	29	386.7	-	-

**Fig. 7:**
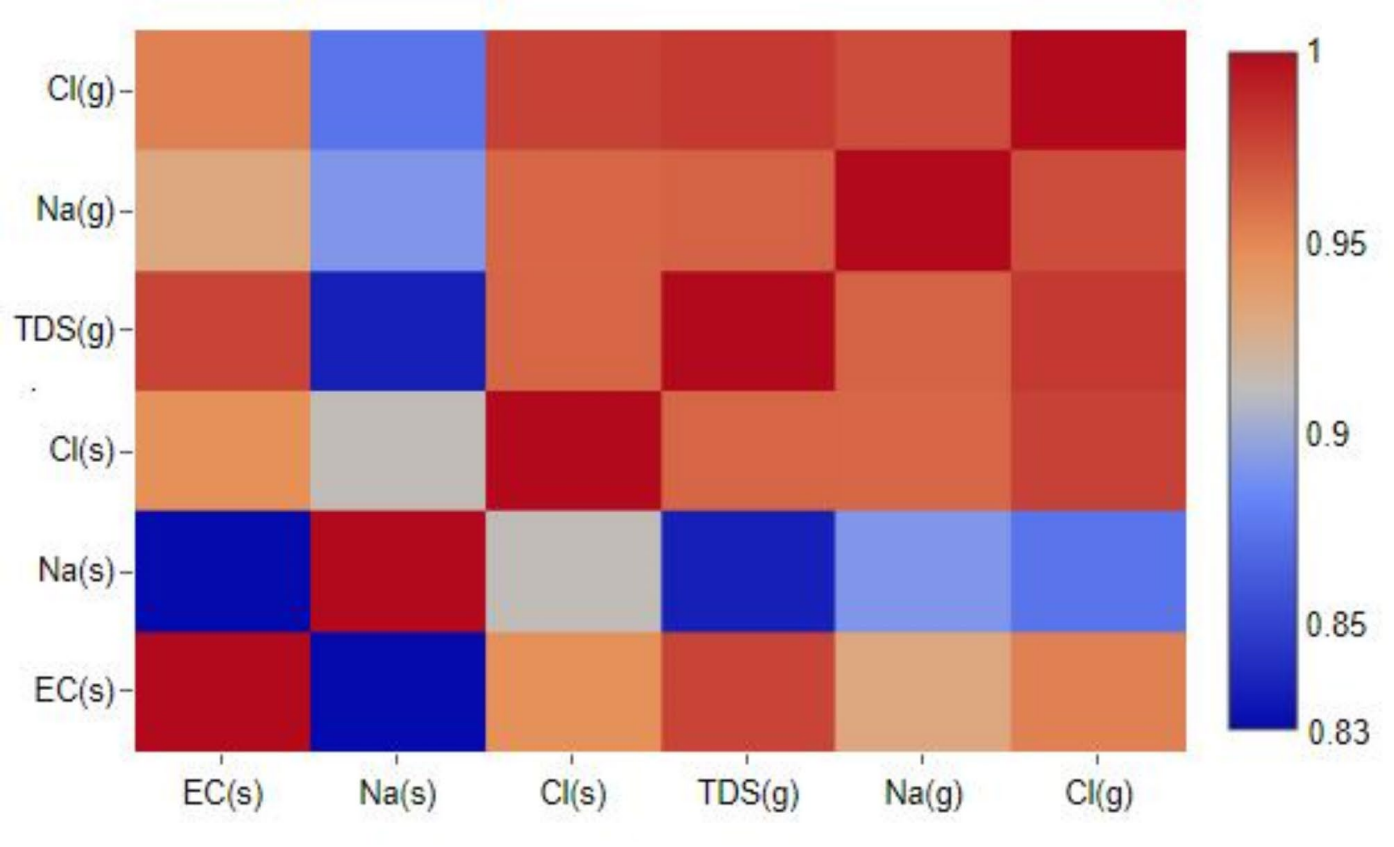
Correlation matrix showing the relationship between soil (s) and groundwater (g) chemical parameters. Strong positive correlations (> 0.83) are observed between electrical conductivity (EC), TDS, sodium (Na^+^), and chloride (Cl^−^) concentrations in soil versus groundwater.

Shallow saline groundwater significantly contributes to soil salinity in the study area. This is clearly shown in the analysis of the correlation coefficient between EC, Na^+^, Cl^−^ in the soil and TDS, Na^+^, Cl^−^ in groundwater, which showed a strong positive relationship (> 0.83), indicating a close relationship between soil salinization and groundwater quality in the study area (Fig. 7).

**Fig. 8:**
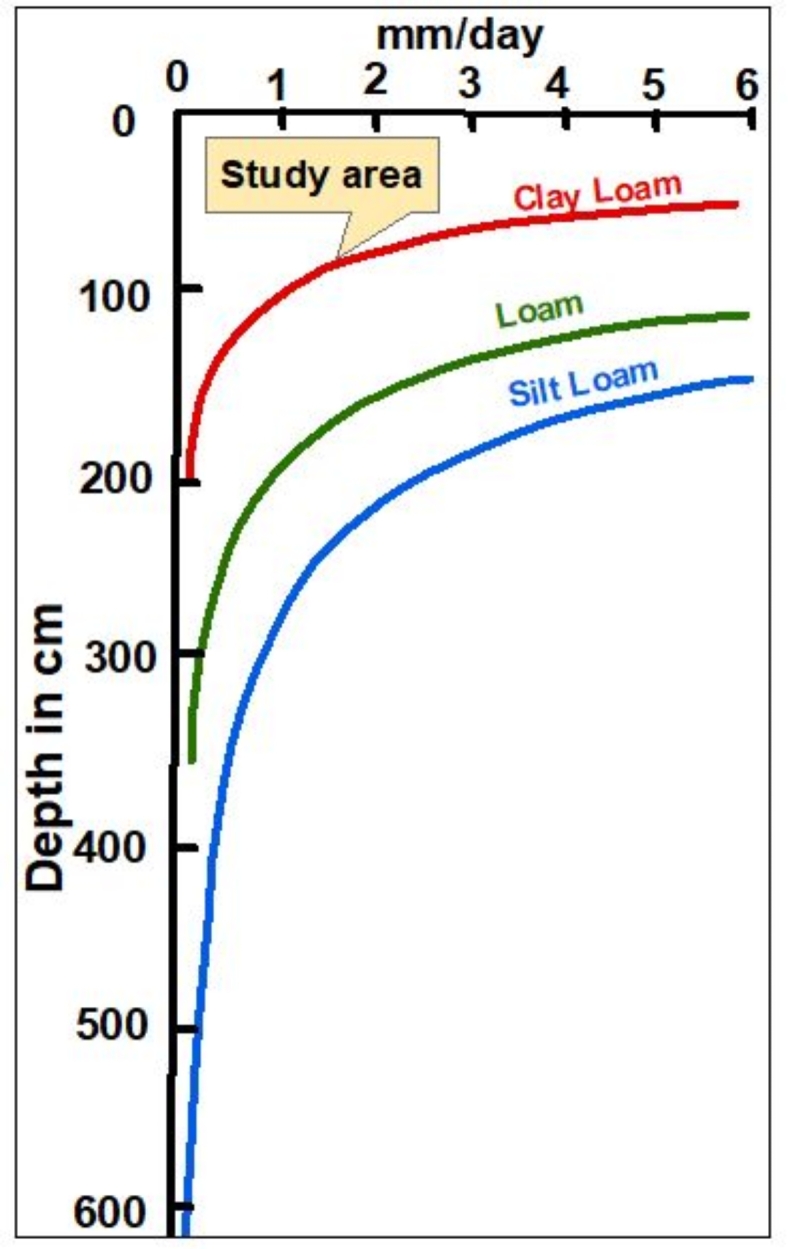
Theoretical relationship between capillary rise velocity and depth to groundwater table for different soil textures  (adapted from^146^). The clay soils and shallow groundwater depths found in the study area (0.5–3 m) suggest maximum capillary flow velocities are likely. This confirms the significant contribution of shallow saline groundwater as a continual source of salts to the soil in the northeastern Nile Delta. .

Soil salinity problems in irrigated agriculture are frequently associated with an uncontrolled, shallow water table within 1–2 m of the surface, allowing capillary transport of salts from the groundwater into the root zone by capillary flow. This rapid salinization process is further exacerbated in irrigated arid climates like the study area, where high evaporation concentrates salts in the soil^144,145^. The rate of soil salt accumulation depends on groundwater depth and salinity, soil texture, and climate^146^. Given the clay-dominated soils and shallow groundwater (from 0.5 to 3 m from the surface) observed in the study area, capillary flow velocity likely operates at maximum possible rates, as shown in Fig. 8. This confirms the significant contribution of groundwater to soil degradation in the northeastern Nile Delta, emphasizing the need to manage the shallow water table and maintain it at a safe depth of at least two meters^110,147^.

### Remediation strategies and recommended further studies

Addressing soil salinization and waterlogging in the northeastern Nile Delta requires a multi-pronged approach targeting water table management, improving irrigation water quality, and soil amendments. A primary focus should be on managing the shallow saline water table, which our study has identified as a key driver of soil degradation in the region. Implementing measures to lower the shallow saline groundwater tables to depths exceeding 2 m is crucial for effectively reducing the capillary rise of salts into the root zone^110,146^. This could be achieved through the enhancement of drainage infrastructure to control the groundwater table at a specified safe depth, coupled with strategic groundwater pumping in affected areas^59,147^.

Concurrent with groundwater management, improving irrigation water quality is essential for maintaining soil health and agricultural productivity in the Delta region. One feasible approach involves blending saline irrigation water with higher-quality freshwater sources to dilute salt and sodium concentrations^114,116^.

Furthermore, addressing the challenges posed by the predominantly clay-textured, low-permeability soils in the study area is critical. Amending these soils with gypsum or organic matter can significantly improve soil structure and infiltration rates^148,149^. The incorporation of crop residues or other organic matter into the field is one of the most effective and accessible methods for enhancing water infiltration in sodic soils. Both surface crop residues and crop root systems contribute to improved soil structure, thereby increasing soil porosity and water infiltration capacity^150,151^.

In addition to these technical interventions, strengthening educational programs and extension services is crucial for promoting the adoption of improved irrigation management practices. This includes facilitating the transition from traditional flood irrigation to more efficient methods such as sprinkler or drip systems^110,152^.

While this study provides valuable insights, several limitations must be acknowledged. The dense population of archaeological sites restricts deep excavations for soil sampling. Future research could benefit from collaboration with local authorities and archaeologists to develop a sampling protocol that accommodates both research needs and heritage preservation, allowing for deeper soil sampling.

Additionally, our sampling was conducted at a single point in time, which may not capture seasonal variations or long-term trends in soil and water quality. Multi-temporal sampling could provide valuable insights into these dynamics. However, given the historical inundation of seawater and Lake Manzala in the study area, the salinity challenges are likely structural rather than seasonal. Therefore, analyses of groundwater salinity across different seasons may consistently reveal high values, indicating that salinity is a chronic issue.

Future research should address these limitations and expand upon our findings. Long-term monitoring studies could elucidate the temporal dynamics of soil degradation processes. Incorporating detailed measurements of soil physical properties and advanced remote sensing techniques could enhance our understanding of the spatial variability of soil degradation. Additionally, exploring the integration of remote sensing and hydrogeological modeling could refine the characterization and simulation of groundwater dynamics in response to management strategies. It is also recommended that future research endeavors adopt a quantitative approach to explore the correlation between different sources of pollutant emissions and variations in irrigation water quality.

## Conclusions

This study presents a comprehensive assessment of soil salinization and waterlogging in the northeastern Nile Delta, a region of profound agricultural and archaeological significance. Our approach incorporated chemical analyses of water and soil samples, water quality index calculations, statistical analyses, and satellite data interpretation.

The findings reveal severe soil degradation, with 42.8% of soil samples classified as strongly saline and 21.4% as very strongly saline. Waterlogging is particularly prevalent in the northern and central zones of the region. We identified three primary drivers of soil degradation: a shallow saline water table, poor-quality irrigation water with high salt and sodium content, and unfavorable soil texture. The Irrigation Water Quality Index (IWQI) indicates that 87.5% of irrigation water samples impose severe restrictions due to elevated salinity and sodium levels. These conditions are especially problematic given that clay-textured soils, which cover 81% of the area, are particularly susceptible to salt accumulation and resistant to effective leaching.

Our analysis demonstrates a strong positive correlation (*r* > 0.83) between shallow saline groundwater and soil salinity, emphasizing groundwater’s crucial role in soil degradation through capillary rise and salt migration. The combined effects of poor irrigation water quality, shallow saline groundwater, and low-permeability soils create a cascade of challenges that severely impact soil health and agricultural productivity. Moreover, these deteriorating conditions threaten the region’s archaeological heritage, as increased salinization and waterlogging accelerate the weathering processes that compromise the structural integrity of archaeological sites.                                                                                                                                                                             Acknowledgements This paper is part of the PhD thesis of the first author. We express our deep appreciation to the Editor and anonymous reviewers for their constructive criticism and valuable feedback, which significantly improved the manuscript. Additionally, we would like to extend our sincere gratitude to Dr. Said A. Shetaia from the Geology Department, Faculty of Science, Al-Azhar University, Cairo, Egypt, for his invaluable assistance throughout this work.

## Electronic Supplementary Material

Below is the link to the electronic supplementary material.

Supplementary Material 1

## Data Availability

The data are available from the corresponding author upon reasonable request.

## References

[1] Zaghloul, E. A., Abdeen, M. M., Elbeih, S. F. & Soliman, M. A. Water logging problems in Egypt’s deserts: Case study Abu Mena archaeological site using geospatial techniques. *Egypt. J. Remote Sens. Space Sci.* **23**(3), 387–399 (2020).

[2] Singh, A. Soil salinization management for sustainable development: A review. *J. Environ. Manage.* **277**, 111383 (2021). 33035935 10.1016/j.jenvman.2020.111383

[3] Han, W., Gong, R., Liu, Y. & Gao, Y. Influence mechanism of salt erosion on the earthen heritage site wall in Pianguan Bastion. *Case Stud. Constr. Mater.* **17**, e01388 (2022).

[4] Polykretis, C. et al. Assessment of water-induced soil erosion as a threat to cultural heritage sites: The case of Chania prefecture, Crete Island, Greece. *Big Earth Data*. **6**(4), 561–579 (2022).

[5] Zhang, Y. Knowledge of earthen heritage deterioration in dry areas of China: Salinity effect on the formation of cracked surface crust. *Herit. Sci.* **11**(1), 41 (2023).

[6] Cisse, E. H. M. et al. Physio-biochemical and metabolomic responses of the woody plant Dalbergia odorifera to salinity and waterlogging. *BMC Plant Biol.* **24**(1), 49 (2024). 38216904 10.1186/s12870-024-04721-5 PMC10787392

[7] Shrivastava, P. & Kumar, R. Soil salinity: A serious environmental issue and plant growth promoting bacteria as one of the tools for its alleviation. *Saudi J. Biol. Sci.* **22**(2), 123–131 (2015). 25737642 10.1016/j.sjbs.2014.12.001 PMC4336437

[8] Pitman, M. G. & Läuchli, A. Global impact of salinity and agricultural ecosystems. *Salinity: Environment-plants-molecules*. **3**, 20 (2002).

[9] Jamil, A., Riaz, S., Ashraf, M. & Foolad, M. R. Gene expression profiling of plants under salt stress. *Crit. Rev. Plant. Sci.* **30**(5), 435–458 (2011).

[10] Tian, L. X. et al. How does the waterlogging regime affect crop yield? A global meta-analysis. *Front. Plant Sci.* **12**, 634898 (2021). 33679848 10.3389/fpls.2021.634898 PMC7933672

[11] Khan, A. A. & Shafiq, M. Water logging and salinization: A serious threat for our agriculture. *Pakistan Geographical Rev.* **58**(1), 2 (2003).

[12] Askri, B., Khodmi, S. & Bouhlila, R. Impact of subsurface drainage system on waterlogged and saline soils in a Saharan palm grove. *Catena*. **212**, 106070 (2022).

[13] Datta, K. K. & De Jong, C. Adverse effect of waterlogging and soil salinity on crop and land productivity in northwest region of Haryana, India. *Agric. Water Manage.* **57**(3), 223–238 (2002).

[14] Singh, A., Krause, P., Panda, S. N. & Flugel, W. A. Rising water table: A threat to sustainable agriculture in an irrigated semi-arid region of Haryana, India. *Agric. Water Manage.* **97**(10), 1443–1451 (2010).

[15] Bouksila, F. et al. Assessment of soil salinization risks under irrigation with brackish water in semiarid Tunisia. *Environ. Exp. Bot.* **92**, 176–185 (2013).

[16] Ding, Z. et al. Seawater intrusion impacts on groundwater and soil quality in the northern part of the Nile Delta, Egypt. *Environ. Earth Sci.* **79**, 1–11 (2020).

[17] Greene, R., Timms, W., Rengasamy, P., Arshad, M. & Cresswell, R. In: Soil and aquifer salinization: Toward an integratedapproachfor salinity management of groundwater. (eds. Jakeman, A.J., Barreteau, O., Hunt, R.J., Rinaudo, JD., Ross, A.)Integrated groundwater management: Concepts, approaches and challenges (377–412). Springer Nature. (2016).

[18] Hagage, M., Madani, A. A. & Elbeih, S. F. Quaternary groundwater aquifer suitability for drinking in Akhmim, Upper Egypt: An assessment using water quality index and GIS techniques. *Arab. J. Geosci.* **15**(2), 196 (2022).

[19] Farid, I., Abbas, M., Bassouny, M., Gameel, A. & Abbas, H. Indirect impacts of irrigation with low-quality water on the environmental safety. *Egypt. J. Soil Sci.* **60**(1), 1–15 (2020).

[20] Amer, R. Spatial relationship between irrigation water salinity, waterlogging, and cropland degradation in the arid and semi-arid environments. *Remote Sens.* **13**(6), 1047 (2021).

[21] Mohanavelu, A., Naganna, S. R. & Al-Ansari, N. Irrigation induced salinity and sodicity hazards on soil and groundwater: An overview of its causes, impacts and mitigation strategies. *Agriculture*. **11**(10), 983 (2021).

[22] Chhabra, R. *Salt-affected Soils and Marginal Waters: Global Perspectives and Sustainable Management* (Springer, 2022).

[23] Khalil, M. M. et al. Poor drainage-induced waterlogging in Saharan groundwater-irrigated lands: Integration of geospatial, geophysical, and hydrogeological techniques. *Catena*. **207**, 105615 (2021).

[24] Elbeih, S. F., Madani, A. A. & Hagage, M. Groundwater deterioration in Akhmim District, Upper Egypt: A remote sensing and GIS investigation approach. *Egypt. J. Remote Sens. Space Sci.* **24**(3), 919–932 (2021).

[25] Vlotman, W. F. Waterlogging and salinity: Cause and effect in the framework of a social, environmental and financial nexus. *Irrig. Sci.* **72**(5), 1283–1290 (2023).

[26] Wang, D. et al. Effects of soil texture on soil leaching and cotton (Gossypium hirsutum l.) growth under combined irrigation and drainage. *Water*. **13**(24), 3614 (2021).

[27] O’Geen, A. T. Soil water dynamics. *Nat. Educ. Knowl.* **4**(5), 9 (2013).

[28] Warrence, N. J., Bauder, J. W. & Pearson, K. E. *Basics of Salinity and Sodicity Effects on Soil Physical Properties* (Departement of Land Resources and Environmental Sciences, Montana State University-Bozeman, 2002).

[29] Tang, S., She, D. & Wang, H. Effect of salinity on soil structure and soil hydraulic characteristics. *Can. J. Soil Sci.* **101**(1), 62–73 (2020).

[30] Ayers, R. S. & Westcot, D. W. *Water Quality for Agriculture* (Food and Agriculture Organization of the United Nations, 1985).

[31] Kotb, T. H., Watanabe, T., Ogino, Y. & Tanji, K. K. Soil salinization in the Nile Delta and related policy issues in Egypt. *Agric. Water Manage.* **43**(2), 239–261 (2000).

[32] Alnaimy, M. A., Shahin, S. A., Vranayova, Z., Zelenakova, M. & Abdel-Hamed, E. M. W. Long-term impact of wastewater irrigation on soil pollution and degradation: A case study from Egypt. *Water*. **13**(16), 2245 (2021).

[33] Yuan, C., Feng, S., Wang, J., Huo, Z. & Ji, Q. Effects of irrigation water salinity on soil salt content distribution, soil physical properties and water use efficiency of maize for seed production in arid Northwest China. *Int. J. Agricultural Biol. Eng.* **11**(3), 137–145 (2018).

[34] Wahba, M., Labib, F. & Zaghloul, A. Impact of the global climate change on land degradation in Egypt. *Int. J. Environ. Pollution Environ. Modelling*. **2**(2), 48–61 (2019).

[35] Hassani, A., Azapagic, A. & Shokri, N. Global predictions of primary soil salinization under changing climate in the 21st century. *Nat. Commun.* **12**(1), 6663 (2021). 34795219 10.1038/s41467-021-26907-3 PMC8602669

[36] Ondrasek, G. & Rengel, Z. Environmental salinization processes: Detection, implications & solutions. *Sci. Total Environ.* **754**, 142432 (2021). 33254867 10.1016/j.scitotenv.2020.142432

[37] Caseldine, C. R. A critical evaluation of soil salinization, waterlogging, and agricultural productive capacity in Hohokam irrigation of the Phoenix Basin, Arizona, USA. *J. Environ. Qual. ***52**(4), 799–813 (2023). 10.1002/jeq2.20486 37148490

[38] Stavi, I., Thevs, N. & Priori, S. Soil salinity and sodicity in drylands: A review of causes, effects, monitoring, and restoration measures. *Front. Environ. Sci.*, **9**, 712831 (2021).

[39] Kumar, P. & Sharma, P. K. Soil salinity and food security in India. *Front. Sustainable Food Syst.* **4**, 533781 (2020).

[40] Arnous, M. O., El-Rayes, A. E. & Green, D. R. Hydrosalinity and environmental land degradation assessment of the East Nile Delta region, Egypt. *J. Coastal. Conserv.* **19**, 491–513 (2015).

[41] AbdelRahman, M. A. & Tahoun, S. GIS model-builder based on comprehensive geostatistical approach to assess soil quality. *Remote Sens. Applications: Soc. Environ.* **13**, 204–214 (2019).

[42] Hammam, A. A. & Mohamed, E. S. Mapping soil salinity in the East Nile Delta using several methodological approaches of salinity assessment. *Egypt. J. Remote Sens. Space Sci.* **23**(2), 125–131 (2020).

[43] AbdelRahman, M. A. et al. Determining the extent of soil degradation processes using trend analyses at a regional multispectral scale. *Land* **12**(4), 855 (2023).

[44] Wang, H. et al. Impacts of long-term saline water irrigation on soil properties and crop yields under maize-wheat crop rotation. *Agric. Water Manage.* **286**, 108383 (2023).

[45] Hagage, M., Abdulaziz, A., Hewaidy, A. & Shetaia, S. Unveiling the past: Utilizing satellite imagery archives to study archaeological landscapes in the northeastern Nile Delta. *Egypt. Anthropocene*. **44**, 100409 (2023).

[46] Ahmed, A. A. & Fogg, G. E. The impact of groundwater and agricultural expansion on the archaeological sites at Luxor, Egypt. *J. Afr. Earth Sc.* **95**, 93–104 (2014).

[47] Kader, R. R., Kafafy, A. H. & El-Sayed, S. S. Identification of groundwater’s type in sarabium archaeological Site–Atfiyah–Egypt and its microbiological effect. *Int. J. Archaeol.* **4**(5), 67–77 (2016).

[48] El-Gohary, M. A. A holistic approach to the assessment of the groundwater destructive effects on stone decay in Edfu temple using AAS, SEM-EDX and XRD. *Environ. Earth Sci.* **75**, 1–11 (2016).

[49] Abudeif, A. M., Aal, A., Masoud, G. Z., Mohammed, A. M. & M. A Detection of groundwater pathways to monitor their level rise in Osirion at Abydos archaeological site for reducing deterioration hazards, Sohag, Egypt using electrical resistivity tomography technique. *Appl. Sci.* **12**(20), 10417 (2022).

[50] El-Hassan, R. A. & Abd El-Tawab, N. A. A multi-analytical study of building materials and deterioration products of the Royal Tombs to Tanis (San El-Hagar). *Int. J. Conserv. Sci.* **14**(4), 1351–1366 (2023).

[51] Hagage, M., Madani, A. A., Aboelyamin, A. & Elbeih, S. F. Urban sprawl analysis of Akhmim city (Egypt) and its risk to buried heritage sites: Insights from geochemistry and geospatial analysis. *Herit. Sci.* **11**(1), 174 (2023).

[52] Vallet, J. M., Hubert-Joly, E., Duberson, S., Leclère, F. & Input of the technical imaging for the study of wall paintings. Example of a lintel (Tomb of King Takelot I at Tanis San El-Hagar, Sharqeya, Egypt). *Egypt. J. Archaeol. Restor. Stud.* **12**(1), 73–87 (2022).

[53] El-Shishtawy, A. M., Atwia, M. G., El-Gohary, A. & Parizek, R. R. Impact of soil and groundwater corrosion on the Hierakonpolis Temple Town archaeological site, Wadi Abu Sufian, Idfu, Egypt. *Environ. Monit. Assess.* **185**, 4491–4511 (2013). 23054264 10.1007/s10661-012-2884-6

[54] Qadir, M., Schubert, S., Ghafoor, A. & Murtaza, G. Amelioration strategies for sodic soils: A review. *Land. Degrad. Dev.* **12**(4), 357–386 (2001).

[55] Peterson, A. M., Helgason, W. H. & Ireson, A. M. How spatial patterns of soil moisture dynamics can explain field-scale soil moisture variability: Observations from a sodic landscape. *Water Resour. Res.* **55**(5), 4410–4426 (2019).

[56] Mohamedin, A. A. M., Awaad, M. S. & Ahmed, A. R. The negative role of soil salinity and waterlogging on crop productivity in the northeastern region of the Nile Delta, Egypt. *Res. J. Agric. Biol. Sci.* **6**(4), 378–385 (2010).

[57] Abuzaid, A. S. Assessing degradation of floodplain soils in north east Nile Delta, Egypt. *Egypt. J. Soil Sci.* **58**(2), 135–146 (2018).

[58] El Nahry, A. H., Ibraheim, M. M., Baroudy, E. & A. A Assessment of soil degradation in the northern part of Nile Delta, Egypt, using remote sensing and GIS techniques. *Egypt. J. Remote Sens. Space Sci.* **11**, 139–154 (2008).

[59] Kaiser, M. F., Rayes, E., Ghodeif, A., Geriesh, K. & B GIS data integration to manage waterlogging problem on the eastern Nile delta of Egypt. *Int. J. Geosci.* **4**(4), 8 (2013).

[60] Abd-Elmabod, S. K., Fitch, A. C., Zhang, Z., Ali, R. R. & Jones, L. Rapid urbanisation threatens fertile agricultural land and soil carbon in the Nile delta. *J. Environ. Manage.* **252**, 109668 (2019). 31604185 10.1016/j.jenvman.2019.109668

[61] Awad, S. R., Fakharany, E. & Z. M Mitigation of waterlogging problem in El-Salhiya area, Egypt. *Water Sci.* **34**(1), 1–12 (2020).

[62] Mohamed, E. S., Belal, A. & Saleh, A. Assessment of land degradation east of the Nile Delta, Egypt using remote sensing and GIS techniques. *Arab. J. Geosci.* **6**, 2843–2853 (2013).

[63] Elbana, M. Negative impacts of improper long-term irrigation using treated wastewater on soil and vegetation performance: Case study in Bahr El-Baqar Drain, Egypt. *EC Agric.* **6**, 17–30 (2020).

[64] El-Amier, Y. A. et al. Hydrochemical assessment of the irrigation water quality of the El-Salam canal, Egypt. *Water* **13**(17), 2428 (2021).

[65] Stanley, D. J. & Warne, A. G. Nile Delta in its destruction phase. *J. Coastal Res.* **14**(3), 795–825. (1998).

[66] Sharqia, A. & Egypt Climate. Retrieved from (2024). https://weatherandclimate.com/egypt/al-sharqia

[67] Sallouma, M. K. Hydrogeological and hydrochemical assess ment of the Quaternary aquifer in the eastern Nile Delta, Egypt. PhD Thesis, 166 (Faculty of Science, Ain Shams University, 1983).

[68] Said, R. *The geological evaluation of the River Nile* (Springer, 1981).

[69] Attwa, M., Gemail, K. & Eleraki, M. Use of salinity and resistivity measurements to study the coastal aquifer salinization in a semi-arid region: A case study in northeast Nile Delta, Egypt. *Environ. Earth Sci.*, **75**, 784. (2016).

[70] El-Aassar, A. H., Hagagg, K., Hussien, R., Oterkus, S. & Oterkus, E. Integration of groundwater vulnerability with contaminants transport modeling in unsaturated zone, case study El-Sharqia, Egypt. *Environ. Monit. Assess.* **195**(6), 1–13 (2023). 10.1007/s10661-023-11298-3 PMC10209242 37225912

[71] Abo-El-Fadl, M. M. Possibilities of groundwater pollution in some areas, East of Nile Delta, Egypt. *Int. J. Environ.* **1**(1), 1–21 (2013).

[72] Eldeeb, H. & Zelenakova, M. Assessment of the economic value of irrigation water considering achieve main crops self-sufficiency: Case study Sharkia Governorate, Egypt. *Sel. Sci. Papers-Journal Civil Eng.* **14**(2), 39–50 (2019).

[73] Forti, L. et al. Geomorphological assessment of the preservation of archaeological tell sites. *Sci. Rep.* **13**(1), 7683 (2023). 37202432 10.1038/s41598-023-34490-4 PMC10195873

[74] Brissaud, P. Les fouilles dans le secteur de la Nécropole royale (1984–1986) in Cahiers de Tanis I. Mission Française des Fouilles de Tanis. *Mémoire de l’institut mauritanien de la. recherche scientifique*, **75**, 7–43. (1987).

[75] Helmi, F. M. & Hefni, Y. K. Nanocomposites for the protection of granitic obelisks at Tanis,Egypt. *Mediterranean Archaeol. Archaeometry* **16**(2), 87–96. (2016).

[76] Kroeper, K. Minshat Abu Omar—burials with palettes. In J Spencer (ed.): *Aspects of Early Egypt*. London: British Museum Press. 70–92 (1996).

[77] Esri ArcGIS Desktop 10.7.1 quick start guide. (2019). https://desktop.arcgis.com/en/quick-start-guides/10.7/arcgis-desktop-quick-start-guide.htm

[78] APHA. *Standard methods for the examination of water and wastewater, 22nd edition edited by E. W. Rice, R. B. Baird, A. D. Eaton and L. S. Clesceri.* Washington, D.C., USA: American Public Health Association (APHA), American Water Works Association (AWWA) and Water Environment Federation (WEF). (2012).

[79] Rodger, B. B., Andrew, D. E. & Eugene, W. R. Standard Methods for the Examination of Water and Wastewater (23nd Edition ed.). Washington: APHA. (2017).

[80] Srinivasan, K. Ion Chromatography Instrumentation for Water Analysis (Chapter 9). Chem. Water . In *Chemistry and Water*; Ahuja, S., Ed.; 329–351 (Elsevier, 2017).

[81] Abdel-Fattah, M. K. Linear regression models to estimate exchangeable sodium percentage and bulk density of salt affected soils in Sahl El-Hossinia, El-Sharkia Governorate, Egypt. *Commun. Soil Sci. Plant Anal.* **50**(16), 2074–2087 (2019).

[82] Richards, L. A. *Diagnosis and Improvement of Saline and Alkali Soils* (US Government Printing Office, 1954).

[83] Klute, A. & Page, A. L. *Methods of Soil Analysis. Part 1. Physical and Mineralogical Methods; Part 2. Chemical and Microbiological Properties* (American Society of Agronomy, Inc, 1986).

[84] Van Reeuwijk, L. International soil reference and information centre. The Netherlands. Variable charges in selected temperate region soils. *Geoderma* **32**, 89–101 (2002).

[85] Sparks, D. L., Page, A. L., Helmke, P. A. & Loeppert, R. H. *Methods of Soil Analysis, Part 3: Chemical Methods*, vol. 14 (Wiley, 2020).

[86] Meireles, A., Andrade, E. M., Chaves, L., Frischkorn, H. & Crisostomo, L. A. A new proposal of the classification of irrigation water. *Revista Ciência Agronômica*. **41**(3), 349–357 (2010).

[87] Ayers, R. S. & Westcot, D. W. Water quality for agriculture. FAO irrigation and drainage paper, 174 pp (Rome, 1985).

[88] Tomaszkiewicz, M., Abou Najm, M. & El-Fadel, M. Development of a groundwater quality index for seawater intrusion in coastal aquifers. *Environ. Model. Softw.* **57**, 13–26 (2014).

[89] Prasad, A. D., Ganasala, P., Hernández-Guzmán, R. & Fathian, F. Remote sensing satellite data and spectral indices: An initial evaluation for the sustainable development of an urban area. *Sustainable Water Resour. Manage.* **8**(1), 19 (2022).

[90] Fisher, A., Flood, N. & Danaher, T. Comparing Landsat water index methods for automated water classification in eastern Australia. *Remote Sens. Environ.* **175**, 167–182 (2016).

[91] Li, S., Ganguly, S., Dungan, J. L., Wang, W. & Nemani, R. R. Sentinel-2 MSI radiometric characterization and cross-calibration with Landsat-8 OLI. *Adv. Remote Sens.* **6**(2), 147 (2017).

[92] Lioumbas, J. et al. Satellite remote sensing to improve source water quality monitoring: A water utility’s perspective. *Remote Sens. Applications: Soc. Environ.* **32**, 101042 (2023).

[93] ESA- European Space Agency. SNAP spurs Earth observation innovation with one million downloads, ESA Applications, 24 May 2022, URL: (2022). https://www.esa.int/Applications/Observing_the_Earth/FutureEO/SNAP_spurs_Earth_observation_innovation_with_one_million_downloads??haha

[94] Liu, H. et al. A comparison of differentwater indices and band downscaling methods for water bodies mapping from Sentinel-2 imagery at 10-M resolution. *Water*. **14**, 2696 (2022).

[95] Edelmann, D., Móri, T. F. & Székely, G. J. On relationships between the Pearson and the distance correlation coefficients. *Stat. Probab. Lett.* **169**, 108960 (2021).

[96] Chakma, A., Bhowmik, T., Mallik, S. & Mishra, U. Application of GIS and geostatistical interpolation method for groundwater mapping. In In Advanced Modelling and Innovations in Water Resources Engineering: Select Proceedings of AMIWRE 2021 (pp. 419–428). Singapore: Springer. (2022).

[97] Elumalai, V., Brindha, K., Sithole, B. & Lakshmanan, E. Spatial interpolation methods and geostatistics for mapping groundwater contamination in a coastal area. *Environ. Sci. Pollut Res.* **24**, 11601–11617 (2017). 10.1007/s11356-017-8681-6 28324252

[98] Metternicht, G. & Zinck, J. A. Spatial discrimination of salt-and sodium-affected soil surfaces. *Int. J. Remote Sens.* **18**(12), 2571–2586 (1997).

[99] Abdelaal, S. M. et al. Mapping spatial management zones of salt-affected soils in arid region: A case study in the east of the Nile Delta. *Egypt. Agron.* **11**(12), 2510 (2021).

[100] AbdelRahman, M. A., Afifi, A. A. & Scopa, A. A time series investigation to assess climate change and anthropogenic impacts on quantitative land degradation in the North Delta, Egypt. *ISPRS Int. J. Geo-Information*. **11**(1), 30 (2022).

[101] Abowaly, M. E. et al. Assessment of soil degradation and hazards of some heavy metals, using remote sensing and GIS techniques in the Northern part of the Nile Delta. *Egypt. Agric.* **13**(1), 76 (2022).

[102] Santos, F., Calle, N., Bonilla, S., Sarmiento, F. & Herrnegger, M. Impacts of soil erosion and climate change on the built heritage of the Pambamarca Fortress Complex in northern Ecuador. *Plos one*, **18**(2), 1–37 (2023). 10.1371/journal.pone.0281869 PMC9949680 36821586

[103] Wild, B., Gerrits, R. & Bonneville, S. The contribution of living organisms to rock weathering in the critical zone. *npj Mater. Degrad.* **6**(1), 98 (2022).

[104] Zewd, I. & Siban, M. The effects of alkalinity on physical and chemical properties of soil. *J. Plant. Biol. Agric. Sci.* **3**, 1–5 (2021).

[105] Shi, J., Long, T., Zheng, L., Gao, S. & Wang, L. Neutralization of industrial alkali-contaminated soil by different agents: Effects and environmental impact. *Sustainability*. **14**(10), 5850 (2022).

[106] Geilfus, C. M. Chloride in soil: From nutrient to soil pollutant. *Environ. Exp. Bot.* **157**, 299–309 (2019).

[107] Li, P. & Du, M. Effect of chloride ion content on pitting corrosion of dispersion-strengthened-high-strength steel. *Corros. Commun.* **7**, 23–34 (2022).

[108] Bronick, C. J. & Lal, R. Soil structure and management: A review. *Geoderma*. **124**(1–2), 3–22 (2005).

[109] Gangwar, P., Singh, R., Trivedi, M. & Tiwari, R. K. Sodic soil: Management and reclamation strategies. In (eds. Shukla, V., Kumar, N.) *Environmental Concerns and Sustainable Development*. 175–190 (Springer, 2020).

[110] Brouwer, C., Goffeau, A. & Heibloem, M. *Irrigation Water Management: Training Manual No. 1-Introduction to Irrigation* (FAO, 1985).

[111] Oudbashi, O. A methodological approach to estimate soil corrosivity for archaeological copper alloy artefacts. *Herit. Sci.* **6**(1), 2 (2018).

[112] Holliday, V. T. *Soils in Archaeological Research* (Oxford University Press, 2004).

[113] Hillel, D., Braimoh, A. K. & Vlek, P. L. *Soil degradation under irrigation*. In: (eds. Braimoh, A. K., Vlek, P. L. G.) Land Use and Soil Resources. Springer. 101–119 (2008).

[114] FAO. *Users Manual for Irrigation with Treated Wastewater* (FAO Regional Office for the Near East, 2003).

[115] Seydehmet, J. et al. Irrigation salinity risk assessment and mapping in arid oasis, Northwest China. *Water* **10**(7), 966 (2018).

[116] Fipps, G. *Irrigation Water Quality Standards and Salinity Management Strategies* (Texas A&M University, 2003).

[117] Łuczak, K., Czerniawska-Kusza, I., Rosik-Dulewska, C. & Kusza, G. Effect of NaCl road salt on the ionic composition of soils and Aesculus hippocastanum L. foliage and leaf damage intensity. *Sci. Rep.* **11**(1), 5309 (2021). 33674734 10.1038/s41598-021-84541-x PMC7935994

[118] Abrol, I. P., Yadav, J. S. & Massoud, F. I. *Salt-affected Soils and Their Management* (Food & Agriculture Org, 1988).

[119] Horneck, D. A., Ellsworth, J. W., Hopkins, B. G., Sullivan, D. M. & Stevens, R. G. *Managing salt-affected soils for crop production*. PNW 601-E. Corvallis, OR, 22 (2007).

[120] Hailu, B. & Mehari, H. Impacts of soil salinity/sodicity on soil-water relations and plant growth in dry land areas: A review. *J. Nat. Sci. Res.* **12**, 1–10 (2021).

[121] El-Defan, A., El-Raies, S., El-Kholy, H. & Osman, A. A summary of water suitability criteria for irrigation. *J. Soil. Sci. Agricultural Eng.* **7**(12), 981–989 (2016).

[122] Singh, A. Soil salinity: A global threat to sustainable development. *Soil Use Manag.* **38**(1), 39–67 (2022).

[123] Lenntech. *Carbonates & bicarbonates hazard of irrigation water. Irrigation Wate*, Accessed 11 November 2023; Retrieved from Lenntech. https://www.lenntech.com/applications/irrigation/bicarbonate/bicarbonate-hazard-of-irrigation-water.htm (2019).

[124] Jacobson, A. T., Bagley, D. M., Dewey, J. C. & Fan, M. Titanium oxyhydroxide–A new effective candidate for resolving the challenging water quality issue of high alkalinity. *J. Environ. Chem. Eng.* **8**(5), 104447 (2020).

[125] Abuzaid, S. Sewage effluent as an alternative source for irrigation: Impact on soil properties and heavy metalstatus. *Annals Agricultural Sci. Moshtohor*. **54**(2), 387–396 (2016).

[126] Abuzaid, A. S. & Jahin, H. S. Implications of irrigation water quality on shallow groundwater in the Nile Delta of Egypt: A human health risk prospective. *Environ. Technol. Innov.* **22**, 101383 (2021).

[127] Abbas, H., Abuzaid, A. S., Jahin, H. & Kasem, D. Assessing the quality of untraditional water sources for irrigation purposes in Al-Qalubiya Governorate, Egypt. *Egypt. J. Soil Sci.* **60**(2), 157–166 (2020).

[128] Taha, A. A., El-Mahmoudi, A. & El-Haddad, I. Pollution sources and related environmental impacts in the new communities southeast Nile Delta, Egypt. *Emirates J. Eng. Res.* **9**(1), 35–49 (2004).

[129] Ramadan, E., Fahmy, M., Nosair, A. & Badr, A. Using geographic information system (GIS) modeling in evaluation of canals water quality in Sharkia Governorate, East Nile Delta, Egypt. *Model. Earth Syst. Environ.* **5**, 1925–1939 (2019).

[130] Zahedi, S. Modification of expected conflicts between drinking water quality index and irrigation water quality index in water quality ranking of shared extraction wells using multi criteria decision making techniques. *Ecol. Ind.* **83**, 368–379 (2017).

[131] Aravinthasamy, P., Karunanidhi, D., Subba Rao, N., Subramani, T. & Srinivasamoorthy, K. Irrigation risk assessment of groundwater in a non-perennial river basin of South India: Implication from irrigation water quality index (IWQI) and geographical information system (GIS) approaches. *Arab. J. Geosci.* **13**, 1–14 (2020).

[132] Al-Hadithi, M., Hasan, K., Algburi, A. & Al-Paruany, K. Groundwater quality assessment using irrigation water quality index and GIS in Baghdad, Iraq. *Jordan J. Earth Environ. Sci.* **10**(1), 15–20 (2019).

[133] Albalasmeh, A. A. et al. Using wastewater in irrigation: The effects on infiltration process in a clayey soil. *Water*. **12**(4), 968 (2020).

[134] Coutellier, V. & Stanley, D. Late Quaternary stratigraphy and paleogeography of the eastern Nile Delta,Egypt. *Mar. Geol.* **77**, 257–275 (1987).

[135] Mansour, B. M. Management of groundwater logging problems along Wadi El-Tumilat, Eastern Nile Delta using mathematical modeling and GIS techniques. 220. M.Sc. thesis, Fac. Science, Suez Canal Univ., Ismailia, Egypt. (2012).

[136] Stanley, J. D. & Clemente, P. L. Increased land subsidence and sea-level rise are submerging Egypt’s Nile Delta coastal margin. *GSA Today*. **27**(5), 4–11 (2017).

[137] WHO, W. *Guidelines for drinking-water quality – 4th ed.* (World Health Organization, 2022). 35417116

[138] US Environmental Protection Agency (USEPA). 2018 edition of the drinking water standards and health advisories tables.US Environmental Protection Agency. (2018).

[139] Abd-Elhamid, H. F. Investigation and control of seawater intrusion in the Eastern Nile Delta aquifer considering climate change. *Water Sci. Technol. Water Supply*. **17**(2), 311–323 (2017).

[140] Mabrouk, M., Jonoski, A., Oude Essink, H. P., hlenbrook, G. & S Impacts of sea level rise and groundwater extraction scenarios on fresh groundwater resources in the Nile Delta Governorates. *Egypt. Water*. **10**(11), 1690 (2018).

[141] Taha, M. S., Armanuos, A. M. & Zeidan, B. A. Creep of Seawater Intrusion in the Nile Delta Aquifer. 13. (2022).

[142] Abd-Elaty, I., Abdoulhalik, A. & Ahmed, A. The impact of future hydrology stresses and climate change on submarine groundwater discharge in arid regions: A case study of the Nile Delta aquifer, Egypt. *J. Hydrology: Reg. Stud.* **47**, 101395 (2023).

[143] Abdelfattah, M. et al. Mapping the impacts of the anthropogenic activities and seawater intrusion on the shallow coastal aquifer of Port Said, Egypt. *Front. Earth Sci.* **11**, 1204742 (2023).

[144] Cui, G., Lu, Y., Zheng, C., Liu, Z. & Sai, J. Relationship between soil salinization and groundwater hydration in Yaoba Oasis, Northwest China. *Water*. **11**(1), 175 (2019).

[145] Lian, H., Sun, Z., Xu, C. & Gu, F. The relationship between the distribution of water and salt elements in arid irrigation areas and soil salination evolution. *Front. Earth Sci.* **10**, 852485 (2022).

[146] Van Hoorn, J. W. Effect of capillary flow on salinization and the concept of critical depth for 1979 determining drain depth. *Paper No. 4.06. In: Proc. International Drainage Workshop, 16–20 May 1978. ILRI Publication No. 25, Wageningen.* 686–700 (1979).

[147] Sojka, M. et al. Sustainable water management in agriculture—The impact of drainage water management on groundwater table dynamics and subsurface outflow. *Sustainability*. **11**(15), 4201 (2019).

[148] Bello, S. K., Alayafi, A. H., Al-Solaimani, S. G. & Abo-Elyousr, K. A. Mitigating soil salinity stress with gypsum and bio-organic amendments: A review. *Agronomy*. **11**(9), 1735 (2021).

[149] Minhas, P. S., Bali, A., Bhardwaj, A. K., Singh, A. & Yadav, R. Structural stability and hydraulic characteristics of soils irrigated for two decades with waters having residual alkalinity and its neutralization with gypsum and sulfuric acid. *Agric. Water Manage.* **244**, 106609 (2021).

[150] Choudhary, O. P., Ghuman, B. S., Thuy, N. & Buresh, R. J. Effects of long-term use of sodic water irrigation, amendments and crop residues on soil properties and crop yields in rice–wheat cropping system in a calcareous soil. *Field Crops Res.* **121**(3), 363–372 (2011).

[151] Leogrande, R. & Vitti, C. Use of organic amendments to reclaim saline and sodic soils: A review. *Arid Land. Res. Manage.* **33**(1), 1–21 (2019).

[152] Kaur, G. et al. Impacts and management strategies for crop production in waterlogged or flooded soils: A review. *Agron. J.* **112**(3), 1475–1501 (2020).

