# Supplementary material for: Monitoring soil salinization and waterlogging in the northeastern Nile Delta linked to shallow saline groundwater and irrigation water quality
Source: Sci Rep. 2024 Nov 13;14:27838. doi: 10.1038/s41598-024-77954-x (PMC11561257; doi:10.1038/s41598-024-77954-x)
Supplement: Supplementary file 1 — Supplementary Material 1 [file 41598_2024_77954_MOESM1_ESM.docx]

Supp Table 1.Irrigation water quality parameter limits and weights for computing IWQI [1,2].

| **WQI parameters** | | **EC**  **(μs/cm)** | **SAR**  **(meq/l)^1/2^** | **Na^+^** | **Cl^-^** | **HCO_3_^-^** |
| --- | --- | --- | --- | --- | --- | --- |
|  |  |  |  | **meq/l** | | |
| **q_i_** | 85-100 | 200≤EC<750 | 2 ≤ SAR° < 3 | 2 ≤ Na < 3 | 1 ≤ Cl < 4 | 1 ≤ HCO_3_ < 1.5 |
|  | 60-85 | 750≤EC<1500 | 3 ≤ SAR° < 6 | 3 ≤ Na < 6 | 4 ≤ Cl < 7 | 1.5 ≤ HCO_3_ < 4.5 |
|  | 35-60 | 1500≤EC<3000 | 6 ≤ SAR° <12 | 6 ≤ Na < 9 | 7 ≤ Cl <10 | 4.5 ≤ HCO_3_ < 8.5 |
|  | 0-35 | EC<200 or  EC≥3000 | SAR° < 2 or  SAR° ≥ 12 | Na < 2 or  Na ≥ 9 | Cl < 1 or  Cl ≥10 | HCO_3_ < 1 or  HCO_3_ ≥ 8.5 |
| **W_i_** | | 0.211 | 0.189 | 0.204 | 0.194 | 0.202 |

Supp Table2. Measured mechanical properties, USDA texture classification, and permeability of collected soil samples.

| NO | clay% | sand% | silt% | Texture class | Permeability |
| --- | --- | --- | --- | --- | --- |
| 1 | 48.8 | 28.8 | 22.4 | Clay | low permeability |
| 2 | 51.3 | 20.4 | 28.3 | Clay | low permeability |
| 3 | 43.4 | 31.35 | 25.25 | Clay | low permeability |
| 4 | 25.35 | 34.1 | 40.55 | Clay Loam | moderately low permeability |
| 5 | 54.7 | 13.7 | 31.6 | Clay | low permeability |
| 6 | 44.55 | 53.4 | 2.05 | Sandy Clay | moderately low permeability |
| 7 | 56.6 | 27.6 | 15.8 | Clay | low permeability |
| 8 | 50.85 | 34.15 | 15 | Clay | low permeability |
| 9 | 54.7 | 13.7 | 31.6 | Clay | low permeability |
| 10 | 4.4 | 90 | 5.6 | Sand | high permeability |
| 11 | 53.67 | 13.13 | 33.2 | Clay | low permeability |
| 12 | 46.4 | 44 | 9.6 | Clay | low permeability |
| 13 | 40.8 | 54.2 | 5 | Sandy Clay | moderately low permeability |
| 14 | 37.2 | 49.3 | 13.5 | Sandy Clay | moderately low permeability |
| 15 | 66.7 | 23.3 | 10 | Clay | low permeability |
| 16 | 62.5 | 25.5 | 12 | Clay | low permeability |
| 17 | 31.9 | 31.6 | 36.5 | Clay Loam | moderately low permeability |
| 18 | 57.7 | 32.8 | 9.5 | Clay | low permeability |
| 19 | 65.5 | 27 | 7.5 | Clay | low permeability |
| 20 | 65.7 | 11 | 23.3 | Clay | low permeability |
| 21 | 79.5 | 12.3 | 8.2 | Clay | low permeability |
| 22 | 64 | 23 | 13 | Clay | low permeability |

**References**

1. Meireles , A., Andrade, E. M., Chaves, L., Frischkorn , H., Crisostomo, L. A. (2010). A new proposal of the classification of irrigation water. *Revista Ciência Agronômica, 41*(3), 349-357.
2. Ayers, R. S., Westcot, D. W. (1999). The water quality in agriculture. *2nd. Campina Grande: UFPB.* (Studies FAO Irrigation and drainage, 29).
